# Inflammatory and endothelial host responses in community-acquired pneumonia: exploring the relationships with HbA1c, admission plasma glucose, and glycaemic gap—a cross-sectional study

**DOI:** 10.3389/fimmu.2024.1372300

**Published:** 2024-05-22

**Authors:** Arnold Matovu Dungu, Agnete Troen Lundgaard, Camilla Koch Ryrsø, Maria Hein Hegelund, Andreas Vestergaard Jensen, Peter Lommer Kristensen, Rikke Krogh-Madsen, Daniel Faurholt-Jepsen, Sisse Rye Ostrowski, Karina Banasik, Birgitte Lindegaard

**Affiliations:** ^1^ Department of Pulmonary and Infectious Diseases, Copenhagen University Hospital - North Zealand, Hilleroed, Denmark; ^2^ Novo Nordisk Foundation Center for Protein Research, Faculty of Health and Medical Sciences, University of Copenhagen, Copenhagen, Denmark; ^3^ Centre for Physical Activity Research, Rigshospitalet, University of Copenhagen, Copenhagen, Denmark; ^4^ Department of Endocrinology and Nephrology, Copenhagen University Hospital - North Zealand, Hilleroed, Denmark; ^5^ Department of Clinical Medicine, Faculty of Health and Medical Sciences, University of Copenhagen, Copenhagen, Denmark; ^6^ Department of Infectious Diseases, Copenhagen University Hospital – Hvidovre Hospital, Hvidovre, Denmark; ^7^ Department of Infectious Diseases, Copenhagen University Hospital – Rigshospitalet, Copenhagen, Denmark; ^8^ Department of Clinical Immunology, Copenhagen University Hospital, Rigshospitalet, Copenhagen, Denmark

**Keywords:** admission p-glucose, community-acquired pneumonia, glycaemic gap, glycated haemoglobin (HbA1c), host response mediators, hyperglycaemia

## Abstract

**Introduction:**

Diabetes is associated with dysregulated immune function and impaired cytokine release, while transient acute hyperglycaemia has been shown to enhance inflammatory cytokine release in preclinical studies. Although diabetes and acute hyperglycaemia are common among patients with community-acquired pneumonia (CAP), the impact of chronic, acute, and acute-on-chronic hyperglycaemia on the host response within this population remains poorly understood. This study investigated whether chronic, acute, and acute-on- chronic hyperglycaemia are associated with distinct mediators of inflammatory, endothelial, and angiogenic host response pathways in patients with CAP.

**Methods:**

In a cross-sectional study of 555 patients with CAP, HbA1c, admission plasma (p)-glucose, and the glycaemic gap (admission p-glucose minus HbA1c- derived average p-glucose) were employed as measures of chronic, acute, and acute-on-chronic hyperglycaemia, respectively. Linear regression was used to model the associations between the hyperglycaemia measures and 47 proteins involved in inflammation, endothelial activation, and angiogenesis measured at admission. The models were adjusted for age, sex, CAP severity, pathogen, immunosuppression, comorbidity, and body mass index. Adjustments for multiple testing were performed with a false discovery rate threshold of less than 0.05.

**Results:**

The analyses showed that HbA1c levels were positively associated with IL-8, IL-15, IL-17A/F, IL-1RA, sFlt-1, and VEGF-C. Admission plasma glucose was also positively associated with these proteins and GM-CSF. The glycaemic gap was positively associated with IL-8, IL-15, IL-17A/F, IL-2, and VEGF-C.

**Conclusion:**

In conclusion, chronic, acute, and acute-on-chronic hyperglycaemia were positively associated with similar host response mediators. Furthermore, acute and acute-on-chronic hyperglycaemia had unique associations with the inflammatory pathways involving GM-CSF and IL-2, respectively.

## 1 Introduction

Community-acquired pneumonia (CAP) is a global public health concern associated with high morbidity and mortality (1, 2). Cytokines and chemokines mediate the inflammatory response in CAP, with excessive responses increasing mortality risk (2–5). Inflammation also involves endothelial activation, which promotes leukocyte migration (6). However, excessive endothelial activation with aberrant angiogenic responses increases the risk of sepsis, septic shock, and mortality in CAP (7–9). Therefore, an improved understanding of factors associated with inflammatory, endothelial, and angiogenic host responses in CAP is crucial to improving care.

Type 2 diabetes, common in CAP (10), is associated with immune cell defects, inflammatory endothelial activation, and hypercoagulability (11). Transient acute hyperglycaemia, assessed by admission p-glucose or the glycaemic gap, is also common in CAP and is associated with mortality (12–14). The glycaemic gap represents the absolute difference between the admission p-glucose and the HbA1c-derived average glucose within the past 3 months (13). This metric quantifies the extent of acute-on-chronic hyperglycaemia by capturing deviations from the baseline, particularly in acute illnesses such as CAP (13). Moreover, transient hyperglycaemia has been shown to induce inflammatory endothelial activation (15) and enhance leukocyte cytokine release in preclinical studies (16), contrasting with the impaired cytokine responses seen in chronic hyperglycaemia as in type 2 diabetes (11). While chronic hyperglycaemia (as seen in type 2 diabetes), acute hyperglycaemia, and acute-on-chronic hyperglycaemia have been linked to dysregulation of the immune host response in COVID-19 pneumonia (17, 18), these associations have been sparingly explored in non-COVID-19 CAP.

Given this, we hypothesised that chronic, acute, and acute-on-chronic hyperglycaemia are associated with distinct inflammatory, endothelial, and angiogenic host response pathways.

The aim of this study was to investigate the associations between chronic, acute, and acute-on-chronic hyperglycaemia and 47 circulating proteins involved in inflammatory, endothelial, and angiogenic host response pathways in patients admitted with CAP.

## 2 Methods

### 2.1 Study design and setting

This study was a cross-sectional study of patients enrolled in the Surviving Pneumonia cohort, a prospective observational cohort study of patients hospitalised with CAP at Copenhagen University Hospital, North Zealand, Denmark (19). The patients enrolled in this study were recruited between January 2019 and November 2021.

Inclusion criteria were adults ≥ 18 years old and hospitalised < 24 h with a new infiltrate on chest x-ray or computed tomography scan, and symptoms (e.g., cough, shortness of breath, and chest pain) or clinical signs (e.g., body temperature ≥38.0°C and abnormal chest examination) consistent with pneumonia.

Exclusion criteria were a positive polymerase chain reaction test for coronavirus disease 2019 (COVID-19) or no admission p-glucose and HbA1c measurement. The study protocol was designed before the COVID-19 pandemic; thus, COVID-19 cases were excluded from the present study. In addition, the roles of chronic, acute, and acute-on-chronic hyperglycaemia as risk factors for dysregulated inflammatory responses in COVID-19 have been described elsewhere (17, 18).

### 2.2 Ethics statement

The study was approved by the Scientific Ethics Committee at the Capital Region of Denmark (H-18024256), registered on ClinicalTrials.gov (NCT03795662), and conducted in accordance with the Declaration of Helsinki (20). Eligible patients were enrolled after oral and written informed consent was obtained. If a patient could not provide consent, the written consent was obtained from a legal guardian or next of kin and an independent physician not part of the study following the guidelines set by the Ethics Committee at the Capital Region of Denmark.

### 2.3 Data collection

Data were prospectively collected during admission through pre-planned patient interviews and medical record reviews. The collected data included demographic information, anthropometry, comorbidities, clinical data, microbiological findings, and laboratory results.

#### 2.3.1 Anthropometry and clinical data

Body mass index (BMI) was calculated using self-reported height and measured weight at admission (Seca, Hamburg, Germany). CAP severity was defined using CURB-65 criteria (21). Data on selected comorbidities (known type 1 and type 2 diabetes, cardiovascular disease, chronic obstructive pulmonary disease, asthma, rheumatological disease, cancer, and immunosuppression) and treatment with glucocorticoids at admission were obtained from the electronic patient file. Immunosuppression was defined as treatment with cancer chemotherapy within the last 28 days, immunosuppressive drugs (e.g., glucocorticoids for more than 14 days corresponding to a prednisolone-equivalent of ≥20 mg/day, monoclonal antibodies used to treat autoimmune and inflammatory diseases), HIV infection, a history of solid organ or bone marrow transplant, or an inborn error of immunity (22).

#### 2.3.2 Microbiology

Microbiological sampling and testing were at the discretion of the attending physicians. Clinically significant microbiological findings in respiratory samples, blood cultures, or pneumococcal/legionella urinary antigen tests were categorised into bacterial, viral, or mixed bacterial–viral aetiology. The cause of CAP was listed as unknown aetiology if the microbiological results were negative or if there was no microbiological sampling.

#### 2.3.3 Definition of chronic, acute, and acute-on-chronic hyperglycaemia

Based on HbA1c measurements obtained at admission and medical history, the study patients were classified according to their chronic hyperglycaemia status, encompassing euglycaemia, prediabetes, undiagnosed diabetes, and known diabetes. Among patients with no prior diabetes diagnosis, HbA1c < 39 mmol/mol (5.7%) was classified as euglycaemia, HbA1c ≥ 39 mmol/mol (5.7%) and < 48 mmol/mol (6.5%) as prediabetes, and HbA1c ≥ 48 mmol/mol (6.5%) as undiagnosed diabetes (10). Known diabetes was based on a prior diabetes diagnosis or treatment with glucose-lowering medication (i.e., metformin, sodium-glucose co-transporter-2 inhibitors, glucagon-like peptide-1 receptor agonists, insulin, and sulfonylureas), regardless of HbA1c level (23). At the time of inclusion, no sodium-glucose co-transporter-2 inhibitors were approved for treating heart failure and kidney disease in people without diabetes.

The definition of acute hyperglycaemia was based on the admission p-glucose level. Euglycaemia: p-glucose < 6.0 mmol/L; mild hyperglycaemia: p-glucose ≥ 6.0 and <11 mmol/L; severe hyperglycaemia: p-glucose ≥ 11.0 mmol/L (10).

Acute-on-chronic hyperglycaemia was determined using the glycaemic gap, calculated by subtracting the admission p-glucose from the HbA1c-derived estimate of the average p-glucose over the previous 2–3 months (13) and categorised in two ways in our analyses. Firstly, the glycaemic gap was categorised such that values above 0 signified the presence of acute-on-chronic hyperglycaemia, while values below 0 indicated the absence of this condition. Secondly, the glycaemic gap was divided into quartiles to provide a granular view of its distribution across our patient cohort (13).

#### 2.3.4 Sampling and technical covariates

Venous blood samples were drawn at admission, collected in EDTA tubes, and centrifuged at 3,000 *g* for 15 min at 4°C. Plasma was stored at −80°C until analysis. Technical covariates relating to the biobank samples included elapsed time between hospital admission and sample collection and total storage time since sample collection. Sine and cosine transformation of the biobank sampling date was used to calculate seasonality, as the cause of CAP varies throughout the year (24).

### 2.4 Assays

The Meso Scale Discovery (MSD) V-PLEX Human Biomarker 54-Plex is based on electrochemiluminescence technology and was used to measure 54 host response mediators (Meso Scale Diagnostics, Maryland, USA). This kit consists of 7 individual multiplex panels. However, we excluded the Th17 panel, which consists of 7 host response mediators, due to quality control issues. The 47 host response mediators in each of the remaining six panels are detailed in **Supplementary Table 1**, while their primary functions and cellular sources are provided in **Supplementary Tables 2**–**5**. Measurements were conducted on the Meso QuickPlex SQ 120 platform (Meso Scale Diagnostics, Maryland, USA) following the manufacturer’s instructions across three different sites in Denmark (**Supplementary Table 1**).

#### 2.4.1 Sample pre-processing

Biomarker concentrations were quantified with the light signal intensity instead of calculated concentrations to avoid noise introduced by standard curves. The sample pre-processing involved removing outliers, correcting batch effects with median normalisation, and transforming the data logarithmically to achieve a normal distribution. Further details about sample pre-processing are provided in the **Supplementary Methods** section.

### 2.5 Dependent variables, exposures, predictors, and covariates

The dependent variables consisted of log-transformed and median-normalised light signal values from 47 proteins, encompassing a wide range of functions, including pro-inflammatory, T cell associated, chemokines, endothelial activation, angiogenesis, and growth factors (**Supplementary Tables 1**–**5**).

In the primary analysis, the predictor variables were HbA1c, admission p-glucose, and the glycaemic gap as measures of chronic, acute, and acute-on-chronic hyperglycaemia, respectively. For the secondary analyses, we used categorised versions of these predictors—diabetes status for chronic hyperglycaemia, acute hyperglycaemia groups, and glycaemic gap quartiles for acute-on-chronic hyperglycaemia. In addition, factors that might affect the quality of the biobank samples (i.e., technical covariates) or might be associated with altered or distinct inflammatory, endothelial, and angiogenic activation (e.g., chronic diseases, pathogen) were included as covariates (7, 25).

### 2.6 Statistical analyses

The continuous variables were summarised as the median with the interquartile range (IQR), while the categorical variables were summarised as counts with percentages.

Principal component analysis (PCA) was conducted on all host response mediators to visualise whether the patients clustered according to the chronic, acute, and acute-on-chronic hyperglycaemia groups.

Linear regression was used to estimate the association between the predictors and the dependent variables. The predictors were evaluated separately to avoid multicollinearity. The associations were first estimated with a base model consisting of each predictor separately, the technical covariates, age, and sex, followed by the adjusted models consisting of base model covariates, microbiology, clinical covariates, and BMI. Model estimates were exponentiated and presented as fold changes with the corresponding 95% confidence intervals.

As detailed in **Supplementary Material**, BMI was missing in 17% of the patients and imputed with the missingness pattern assumed to be missing at random. The adjusted model estimates from each imputed dataset were combined with Rubin’s rules and compared to model estimates from complete case analyses. Each model was checked for linearity, homogeneity of variance, normality of residuals, and absence of multicollinearity.

Correction for multiple testing was performed using the Benjamini–Hochberg method with a false discovery rate threshold of 0.05. All statistical comparisons were two-sided, with *p* < 0.05 considered significant. The statistical analyses were performed with R software (R version 4.0.3), and multiple imputations were performed using the R package MICE.

## 3 Results

### 3.1 Baseline characteristics

**Figure 1:**
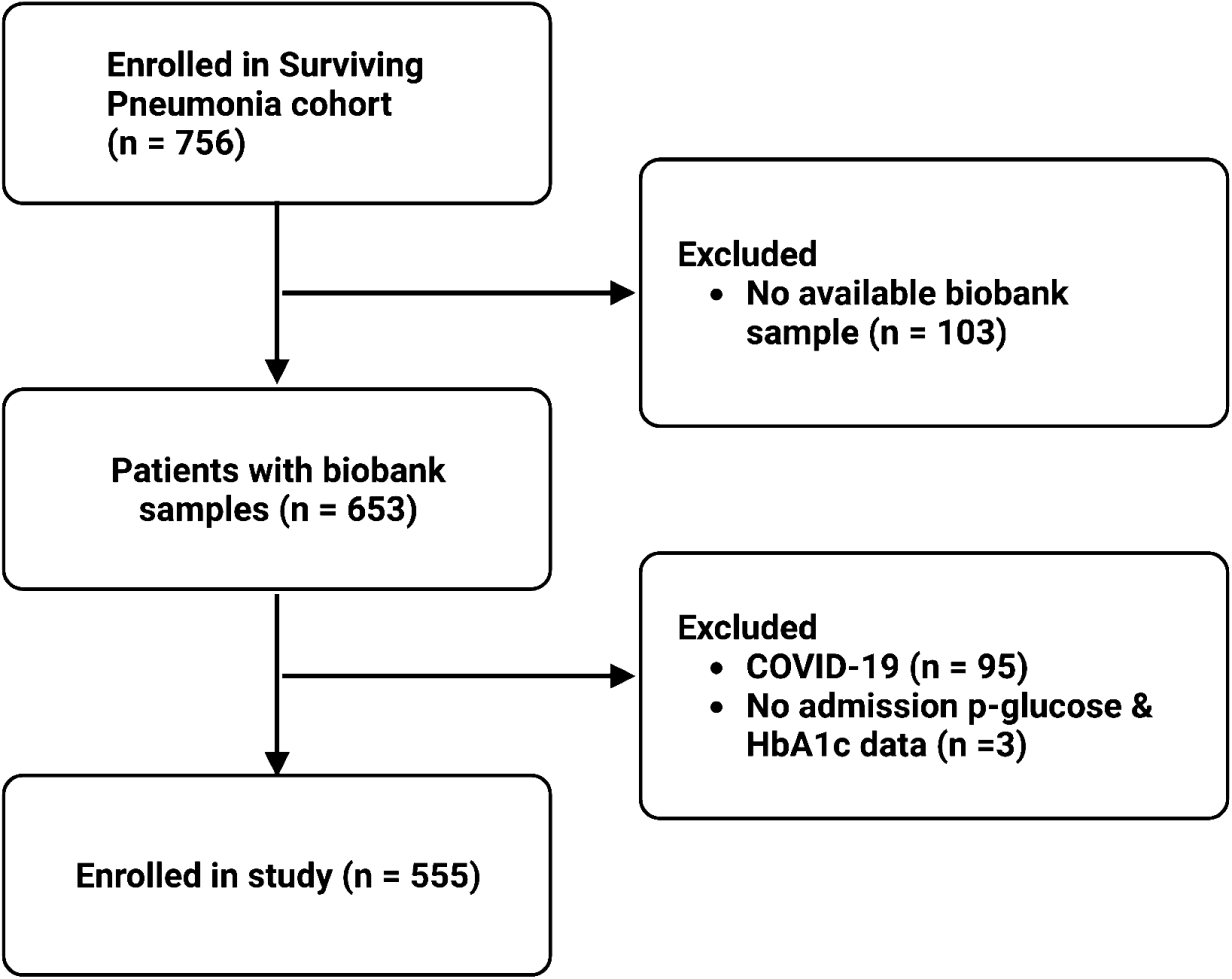
Flowchart of study enrolment. COVID-19, coronavirus disease 2019.

Among 756 patients enrolled in the Surviving Pneumonia cohort during the study period, 653 had an available biobank sample. Ninety-eight patients were excluded due to a positive COVID-19 test (*N* = 95) or the absence of HbA1c or admission p-glucose samples (*N* = 3), leaving 555 patients for inclusion in this study (**Figure 1**). Patient characteristics are presented in **Table 1**. The median age was 74 years (IQR: 64, 81), 268 (48%) were women, 196 (35%) had prediabetes, 22 (4.0%) had undiagnosed diabetes, and 101 (18%) had known diabetes. Admission p-glucose concentrations were classified as normal (<6 mmol/L) in 100 (18%) patients, mild (6-11 mmol/L) in 390 (70%) patients, and severe (≥11 mmol/L) in 65 (12%) patients. The glycaemic gap was > 0 mmol/mol in 360 (65%) patients.

**Table 1:** Patient characteristics.

Characteristic	*N*	*N* = 555
Demography		
Age (years), median [IQR]	555	74 [64, 81]
Female sex, *n* (%)		268 (48%)
Body mass index (kg/m^2^), median [IQR]	458	26 (22, 30)
Comorbidities, *n* (%)		
Chronic hyperglycaemia groups	555	
Euglycaemic		236 (43)
Prediabetes		196 (35)
Undiagnosed diabetes mellitus		22 (4.0)
Known diabetes mellitus		101 (18)
Chronic obstructive pulmonary disease (yes)	555	205 (37)
Asthma (yes)	555	70 (13)
Cardiovascular disease (yes)	555	137 (25)
Cancer (yes)	555	104 (19)
Rheumatic disease (yes)	555	35 (6.3)
Immunosuppressed (yes)	555	57 (10.3)
Glucocorticoids at admission (yes)	555	197 (35)
Glycaemic variables		
HbA1c (mmol/mol), median [IQR] HbA1c (%), median [IQR]	555	40 [36, 45] 6.7 [6.1, 7.4]
Admission p-glucose (mmol/L), median [IQR]	555	7.21 [6.28, 8.68]
Acute hyperglycaemia groups, *n* (%)	555	
Normal (admission p-glucose < 6 mmol/L)		100 (18)
Mild (admission p-glucose 6–11 mmol/L)		390 (70)
Severe (admission p-glucose ≥ 11 mmol/L)		65 (12)
Glycaemic gap (mmol/L), median [IQR]	555	0.54 [−0.36, 1.86]
Acute-on-chronic hyperglycaemia groups, *n* (%)	555	
Glycaemic gap > 0 mmol/L		360 (65)
Glycaemic gap < 0 mmol/L		195 (35)
Disease severity and microbial aetiology, *n* (%)		
CURB-65 score	555	
Mild, 0–1		299 (54)
Moderate, 2		185 (33)
Severe, 3–5		71 (13)
Microbial aetiology	555	
Unknown pathogen		359 (65)
Bacterial		143 (26)
Mixed bacterial and viral		14 (2.5)
Viral		39 (7.0)

The CURB-65 score is based on five variables: confusion, urea >7 mmol/L, respiratory rate ≥30 breaths/min, systolic blood pressure <90 mmHg or diastolic blood pressure ≤60 mmHg, and age ≥65 years.

A pathogen was identified in 196 (35%) patients. The most frequently isolated pathogens were *Haemophilus influenzae, Streptococcus pneumoniae, Staphylococcus aureus, Legionella pneumophila*, and influenza A virus (**Supplementary Figure 1**).

### 3.2 Clustering according to chronic, acute, and acute-on-chronic hyperglycaemia

The PCA plots of the first and second principal components revealed no distinct clustering of host response pathways in the 47 host response mediators according to chronic (**Supplementary Figure 2A**), acute (**Supplementary Figure 2B**), or acute-on-chronic (**Supplementary Figure 2C**) hyperglycaemia groups.

### 3.3 Associations between chronic hyperglycaemia and host response pathways

**Figure 2:**
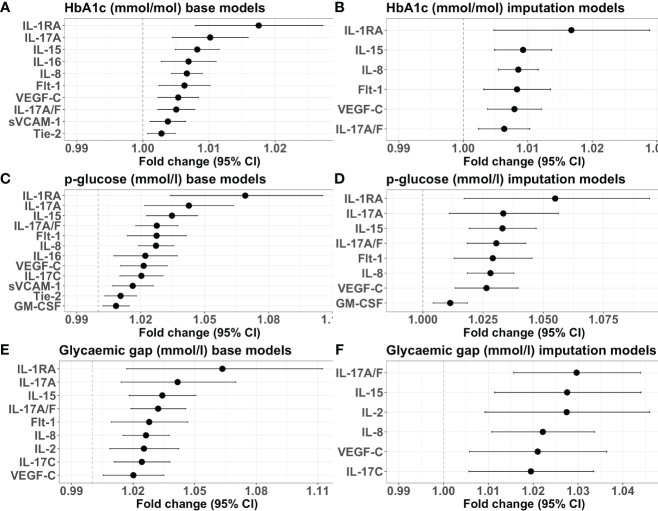
Forest plots illustrating the associations in base and adjusted models. **(A)** Base models with HbA1c as the predictor. **(B)** Adjusted models with HbA1c as the predictor. **(C)** Base models with admission p-glucose as the predictor. **(D)** Adjusted models with admission p-glucose as the predictor. **(E)** Base models with the glycaemic gap as the predictor. **(F)** Adjusted models with the glycaemic gap as the predictor. The x-axis shows the fold change in protein concentration for each 1 unit increase in HbA1c (mmol/mol), admission p-glucose (mmol/L), or glycaemic gap (mmol/L), with the corresponding 95% confidence intervals. Only proteins with statistically significant false discovery rate-adjusted p-values are included.

In the base models, HbA1c was positively associated with concentrations of the chemokine IL-8, proinflammatory cytokines IL-15 and IL-16, T helper (Th)17 cytokine IL-17A/F, anti-inflammatory cytokine IL-1RA, endothelial activation marker soluble vascular cell adhesion molecule-1 (sVCAM1), and proteins involved in angiogenesis: vascular endothelial growth factor-C (VEGF-C), soluble fms-like tyrosine kinase-1 (sFlt-1), and soluble tyrosine kinase with immunoglobulin-like and EGF-like domains 2 (sTie-2) (**Figure 2A**).

In the adjusted models, the positive associations between HbA1c and IL-8, IL-15, IL-17A/F, IL-1RA, VEGF-C, and sFlt-1 remained significant (**Figure 2B**).

HbA1c was not associated with other proteins, including the pro-inflammatory cytokines IL-6 and tumour necrosis factor-alpha (TNF-α) (**Supplementary Figure 3**). The model estimates remained consistent when results using imputed data and complete case analyses were compared (**Supplementary Figures 4**, **5A**).

In the secondary analyses, there were no associations between diabetes status and the host response mediators in base (**Supplementary Table 6**) and adjusted models (**Supplementary Table 7**).

### 3.4 Associations between acute hyperglycaemia and host response pathways

In the base models, admission p-glucose was positively associated with the haemopoietic growth factor granulocyte-macrophage colony-stimulating factor (GM-CSF), IL-8, IL-15, IL-16, IL-1RA, IL-17A/F, IL-17A, IL-17C, sVCAM-1, VEGF-C, sFlt-1, and sTie-2 (**Figure 2C**).

The positive associations between admission p-glucose and IL-8, IL-15, IL-17A/F, IL-17A, IL-1RA, GM-CSF, VEGF-C, and sFlt-1 remained significant in the adjusted analyses (**Figure 2D**).

No associations between admission p-glucose and other proteins were found (**Supplementary Figure 6**). When comparing estimates from fully adjusted models using imputed data and complete case analysis, IL-17A, sFlt-1, and IL-1RA were significant only in analyses using imputed data (**Supplementary Figures 7**, **5B**).

The secondary analyses found no associations between the acute hyperglycaemia groups and the host response mediators in the base (**Supplementary Table 8**) and adjusted models (**Supplementary Table 9**).

### 3.5 Associations between acute-on-chronic hyperglycaemia and host response pathways

In the base models, the glycaemic gap was positively associated with concentrations of the proinflammatory cytokine IL-2, IL-8, IL-15, IL-1RA, IL-17A/F, IL-17A, IL-17C, sFlt-1, and VEGF-C (**Figure 2E**).

In the adjusted models, the positive associations between the glycaemic gap and IL-2, IL-8, IL-15, IL-17A/F, IL-17C, and VEGF-C remained significant (**Figure 2F**).

No associations between the glycaemic gap and other proteins were observed (**Supplementary Figure 8**). When comparing estimates from fully adjusted models using imputed data and complete case analysis, IL-17C and VEGF-C were found significant only in analyses using imputed data (**Supplementary Figures 9**, **5C**).

In models with the glycaemic quartiles as the predictors, no associations were found between acute-on-chronic hyperglycaemia and the host response mediators in base (**Supplementary Table 10**) and adjusted models (**Supplementary Table 11**).

## 4 Discussion

### 4.1 Main findings

HbA1c, admission p-glucose, and the glycaemic gap were positively associated with overlapping inflammatory, endothelial, and angiogenic host response pathways. The main findings of the present study are as follows: (1) chronic hyperglycaemia (HbA1c), acute hyperglycaemia (admission p-glucose), and acute-on-chronic hyperglycaemia (glycaemic gap) were positively associated with the chemokine IL-8, pro-inflammatory cytokine IL-15, Th17 cytokine IL-17A/F, and lymphangiogenic growth factor VEGF-C; (2) chronic and acute hyperglycaemia were positively associated with the anti-inflammatory cytokine IL-1RA and the anti-angiogenic decoy receptor sFlt1; (3) acute hyperglycaemia was positively associated with the haematopoietic growth factor and proinflammatory cytokine GM-CSF; and (4) acute-on-chronic hyperglycaemia was positively associated with the pro-inflammatory cytokine IL-2.

The secondary analyses found no associations between the three hyperglycaemia measures entered as categorical variables and the host response mediators after adjustment for multiple testing.

### 4.2 Glycaemic measures

The immune response to CAP is a complex process of innate and adaptive immunity mediated by pro-inflammatory responses, endothelial activation, and pneumonia resolution mediated by anti-inflammatory responses (2, 26, 27). Preclinical *in vitro* and *in vivo* studies have shown that acute hyperglycaemia induces proinflammatory cytokine and chemokine responses and endothelial activation (28–30), while type 2 diabetes is associated with dysregulated immune responses and endothelial dysfunction (11). Currently, data on the impact of acute, chronic, and acute-on-chronic on the host response in CAP remain limited, highlighting a gap in our understanding of how chronic and acute glucometabolic dysregulation influences CAP immunopathogenesis.

We used various glycaemic measures to investigate the associations of chronic, acute, and acute-on-chronic hyperglycaemia with host response pathways. HbA1c concentrations provide insight into glycaemic control over the preceding 2–3 months and are unaffected by the acute stress response induced by acute illness (31), while the admission p-glucose level encompasses chronic hyperglycaemia and stress-induced hyperglycaemia during infection (32). In addition, by utilising HbA1c-adjusted glycaemic variables, such as the glycaemic gap, we were able to explore the impact of acute-on-chronic hyperglycaemia itself, minimising potential interference from long-term hyperglycaemia (13).

### 4.3 Common associations of chronic, acute, and acute-on-chronic hyperglycaemia with host response pathways

The observation that all three measures of hyperglycaemia in our study were associated with IL-8, IL-15, IL-17, and VEGF-C suggests that these chemokine, cytokine, and lymphangiogenic pathways are influenced by chronic hyperglycaemia rather than solely acute and acute-on-chronic hyperglycaemia.

To our knowledge, our study is the first to show that chronic, acute, and acute-on-chronic hyperglycaemia are positively associated with the mediators of innate immunity IL-8 and IL-15 in patients with CAP. IL-8 is a central mediator of innate immunity as a chemotactic factor and activator of neutrophils (33). In patients with CAP, IL-8 has been positively associated with increased risk of mortality (34). During acute inflammation, IL-15 plays a crucial role in bridging innate and adaptive immunity by regulating the activation, proliferation, and survival of T cells and natural killer (NK) cells and enhancing neutrophil IL-8 secretion (35). Conceptually, our findings are consistent with prior research that showed that chronic hyperglycaemic conditions, such as type 2 diabetes mellitus, elevated HbA1c levels, and insulin resistance, are associated with higher concentrations of IL-8 (36) and IL-15 (37). However, the role of IL-15 in chronic hyperglycaemia is multifaceted, with animal studies associating it with positive glucometabolic effects, including reduced fat mass, decreased insulin resistance, and reduced adipose tissue inflammation (38).

Furthermore, our findings are in line with a study in individuals without diabetes mellitus that reported an increase in systemic IL-8 levels during an oral glucose tolerance test, with a positive correlation between IL-8 and post-load glucose levels and a more pronounced IL-8 increase in individuals with impaired glucose tolerance (30). Similar data regarding increased concentration of IL-15 during transient hyperglycaemia are lacking.

Cellular adaptive immunity is mediated by cytokines secreted by T cells (39). To our knowledge, our study is the first to describe that chronic, acute, and acute-on-chronic hyperglycaemia are positively associated with cytokines in the IL-17 family in patients with CAP. The cytokines in the IL-17 family are secreted by the adaptive T helper 17 cells and mediate the immune response to extracellular pathogens and essential for mucosal immunity (39). Although crucial for adaptive immunity, increased IL-17 concentrations have been associated with greater disease severity, higher mortality rates, and an increased risk of ICU admission in patients with CAP (5).

The cytokines in the IL-17 family are secreted by the adaptive T helper (Th) 17 cells and mediate the immune response to extracellular pathogens and essential for mucosal immunity (39).

Our results regarding chronic hyperglycaemia are consistent with previous research showing that individuals with type 2 diabetes mellitus exhibit elevated levels of Th17 cell subsets and IL-17 concentrations (40, 41). IL-17 is thought to be involved in the pathogenesis of type 2 diabetes by inducing adipose tissue inflammation and upregulation of pro-inflammatory cytokines that induce insulin resistance and β-cell failure (42). However, evidence is scarce on whether short-term acute hyperglycaemia can stimulate IL-17 production in humans. In contrast, research using mouse models of autoimmune diseases indicates that high glucose consumption stimulates Th17 cell generation and exacerbates Th17-driven autoimmune responses (43).

Endothelial and angiogenic responses are essential for the migration of leukocytes from blood vessels to inflammation sites (6). Our study is the first, to our knowledge, to show that chronic, acute, and acute-on-chronic hyperglycaemia are positively associated with VEGF-C.

The main function of VEGF-C is to promote the proliferation, migration, and survival of lymphatic endothelial cells (44). Currently, the prognostic significance of VEGF-C in patients with CAP remains unexplored. Furthermore, the positive association between chronic hyperglycaemia and VEGF-C are similar to previous findings from a study that found the HbA1c and fasting p-glucose were positively correlated with VEGF-C levels (30). Furthermore, hyperglycaemic conditions have been shown to increase VEFG-C expression *in vitro* (45).

Thus, our findings suggest that chronic, acute, and acute-on-chronic hyperglycaemia might be associated with enhanced host response mediators of innate and adaptive immunity and lymphatic function.

### 4.4 Common associations of chronic and acute hyperglycaemia with host response pathways

Anti-inflammatory responses during CAP are essential for CAP resolution and prevention of immune-mediated tissue damage (2). To our knowledge, our study is the first to show that chronic and acute hyperglycaemia were positively associated with the anti-inflammatory cytokine IL-1RA (39). IL-1RA mediates the anti-inflammatory response by binding to the receptors of the pro-inflammatory cytokines of IL-1α and IL-1β (39), which induce fever, the acute phase response, proinflammatory cytokine production, upregulation of endothelial adhesion molecules, and T- and B-cell proliferation, to name the most essential (46). However, high IL-1RA concentrations are associated with an increased risk of mortality in CAP (47). Regarding chronic hyperglycaemia, our findings are consistent with previous research showing that IL-1RA concentrations are elevated in conditions with impaired glucose tolerance and type 2 diabetes, presumably as an anti-inflammatory response to adipose tissue inflammation induced by IL-1α and IL-1β secreted by macrophages (48).

Furthermore, our findings are consistent with a previous study that showed that acute hyperglycaemia was associated with higher IL-1RA concentrations in mechanically ventilated patients with CAP and sepsis (49).

Furthermore, our study is the first to suggest that chronic and acute hyperglycaemia might also impact antiangiogenic processes in CAP, considering the association with sFlt-1, a decoy receptor for angiogenic growth factors like VEGF-A, VEGF-B, and placental growth factor (50). Our findings thus align with previous research that has shown that sFlt-1 and VEGFs are elevated in chronic hyperglycaemic states, such as impaired glucose tolerance and type 2 diabetes (51, 52). In chronic hyperglycaemia, elevated sFlt-1 is thought to counteract the abnormal angiogenesis mediated by VEGFs (51, 52). On the other hand, the relationship between transient acute hyperglycaemia and sFlt-1 levels remains unclear, as does the impact of sFlt-1 on the prognosis of patients with CAP.

Notably, in the adjusted analyses, chronic and acute hyperglycaemia were not associated with sVCAM-1, an endothelial activation marker, or Tie-2, which regulates angiogenesis. These findings might be explained by adjusting for chronic diseases associated with higher levels of sVCAM-1 and sTie-2 and endothelial injury, such as diabetes and cardiovascular disease (53, 54).

Our findings suggest that chronic and acute hyperglycaemia might play a role in the anti-inflammatory responses and modulation of angiogenic processes in patients with CAP.

### 4.5 Associations between acute hyperglycaemia and host response pathways

Acute hyperglycaemia was uniquely associated with the haematopoietic growth factor GM-CSF. A similar association has, to our knowledge, not been previously described in patients with CAP. GM-CSF plays a crucial role in innate immunity by promoting the production, maturation, and inflammatory activation of myeloid cells, such as monocytes and neutrophils (55). Another essential function of GM-CSF during the inflammatory response is to repair damaged alveolar epithelium by expanding the alveolar epithelial cells (55). In preclinical studies, GM-CSF has been shown to be protective in animal models of viral and bacterial pneumonia, and GM-CSF has been tested as a therapeutic in patients with alveolar damage due to acute respiratory distress syndrome (56).

The distinct association between acute hyperglycaemia and GM-CSF within the context of CAP is intriguing yet unclear. Nonetheless, our findings align with a study that showed a significant increase in GM-CSF production during acute hyperglycaemia during a hyperglycaemic clamp, with p-glucose levels clamped at 9–11 mmol/L, compared to a euglycaemic clamp with p-glucose clamped at 4–6 mmol/L (57). Thus, our findings suggest an association between acute hyperglycaemia and myeloid cell production in CAP.

### 4.6 Associations between acute-on-chronic hyperglycaemia and host response pathways

To the best of our knowledge, our study is the first to identify an association between acute-on-chronic hyperglycaemia and IL-2 in CAP. IL-2 is primarily produced by activated T cells, and it plays a crucial role in the differentiation and function of Th cell effector subsets, cytotoxic T cells, anti-inflammatory T regulatory (Treg) cells, and NK cells (58). Furthermore, our results align with *in vitro* findings where incubation of CD4+ cells in escalating glucose concentrations led to progressively higher activation of the IL-2 pathway (59).

### 4.7 Negative findings

It was surprising that we did not observe an association between chronic hyperglycaemia and many inflammatory mediators previously associated with chronic hyperglycaemia or type 2 diabetes in non-acutely ill individuals, such as CRP; the pro-inflammatory cytokines IL-1α, IL-6, and TNF-α (47, 48); or the chemokines eotaxin, macrophage inflammatory protein-1α, interferon-gamma inducible protein-10, and monocyte chemoattractant protein-1 (36).

Similar to the largest study (*n* = 1,895) examining the association between chronic hyperglycaemia stratified by diabetes diagnosis and the inflammatory host response in CAP, we did not find an association between chronic hyperglycaemia and IL-6, TNF-α, or the anti-inflammatory cytokine IL-10 (49). Our study adds to these findings by evaluating the association between chronic hyperglycaemia estimated using HbA1c and inflammatory host response.

One possible explanation is that the intense acute inflammatory response during CAP might mask or outweigh the low-grade inflammatory effects of chronic hyperglycaemia. However, considering the specific associations we found between chronic and other inflammatory mediators, such as the IL-17 cytokine family, chronic hyperglycaemia might be associated with activating specific inflammatory pathways in CAP.

### 4.8 Perspectives

The associations between the three hyperglycaemic measures and the host response mediators observed in this study are not yet understood, highlighting the need for additional research. While acute and acute-on-chronic hyperglycaemia are recognised as risk factors for short-term mortality, diabetes is associated with increased long-term mortality in CAP (12). However, whether the host response mediators identified in this study as associated with chronic, acute, and acute-on-chronic hyperglycaemia mediate the increased CAP mortality risk associated with hyperglycaemia remains an open question.

### 4.9 Strengths and limitations

Our study is the first to investigate the association of chronic, acute, and acute-on-chronic with inflammatory, endothelial, and angiogenic host responses in CAP. We sought to minimise information loss and avoid arbitrary cutoff values, enhance statistical power, and increase the precision of effect estimation by estimating the predictors as continuous variables in our primary analysis.

However, our study has some limitations. We acknowledge that the design of our observational study precludes the ability to establish causality. Additionally, the current body of research limits our capacity to provide a detailed mechanistic explanation for the association of the three hyperglycaemia measures with different sets of host response mediators. These limitations highlight the preliminary nature of our findings and the need for further research. Using raw signal values to estimate the protein concentrations in patients with CAP is novel, and its validity in this population requires further investigation. Although we performed extensive adjustments, residual confounding cannot be ruled out. Moreover, 17% of patients had missing BMI data, which were imputed. However, the directions of the associations were similar when analyses performed with imputed data and complete case analyses were compared. Nevertheless, some associations with admission p-glucose and the glycaemic gap as predictors that were significant in analyses with imputed data were not significant in complete case analyses, probably due to lack of power. Even though we performed extensive adjustment for potential confounders, we cannot rule out residual confounding.

### 4.10 Conclusions

Our findings indicate that chronic, acute, and acute-on-chronic hyperglycaemia were associated with the activation of similar inflammatory, endothelial, and angiogenic host response pathways in patients with CAP. The exceptions were the associations between acute hyperglycaemia and GM-CSF and between acute-on-chronic hyperglycaemia and IL-2, suggesting that acute and acute-on-chronic hyperglycaemia might activate specific inflammatory pathways.

## Data availability statement

The datasets used for the current study are not publicly available. However, pseudonymised data can be provided by the corresponding author upon a reasonable request.

## Ethics statement

The studies involving humans were approved by Scientific Ethics Committee at the Capital Region of Denmark. The studies were conducted in accordance with the local legislation and institutional requirements. The participants provided their written informed consent to participate in this study.

## Author contributions

AD: Conceptualization, Data curation, Formal analysis, Methodology, Project administration, Visualization, Writing – original draft, Writing – review & editing. AL: Formal analysis, Methodology, Supervision, Writing – review & editing. CR: Data curation, Writing – review & editing. MH: Data curation, Writing – review & editing. AJ: Conceptualization, Supervision, Writing – review & editing. PK: Conceptualization, Supervision, Writing – review & editing. RK-M: Conceptualization, Supervision, Writing – review & editing. DF: Conceptualization, Data curation, Supervision, Writing – review & editing. SO: Data curation, Writing – review & editing. KB: Data curation, Formal Analysis, Writing – review & editing. BL: Conceptualization, Funding acquisition, Project administration, Supervision, Writing – review & editing.

## Glossary

**Table:**

bFGF	basic fibroblast growth factor
BMI	body mass index
CAP	community-acquired pneumonia
COPD	chronic obstructive pulmonary disease
COVID-19	coronavirus disease 2019
CRP	C-reactive protein
Eotaxin	eotaxin
Eotaxin-3	eotaxin-3
GM-CSF	granulocyte-macrophage colony-stimulating factor
HbA1c	glycated haemoglobin
ICAM-1	intercellular adhesion molecule-1
IFN-γ	interferon-gamma
IL-1α	interleukin-1α
IL-1β	interleukin-1β
IL-1RA	interleukin-1 receptor antagonist
IL-2	interleukin-2
IL-3	interleukin-3
IL-4	interleukin-4
IL-5	interleukin-5
IL-6	interleukin-6
IL-7	interleukin-7
IL-8	interleukin-8
IL-9	interleukin-9
IL-10	interleukin-10
IL-12/IL-23p40	interleukin-12/interleukin-23p40
IL-12p70	interleukin-12p70
IL-13	interleukin-13
IL-15	interleukin-15
IL-16	interleukin-16
IL-17A	interleukin-17A
IL-17A/F	interleukin-17A/F
IL17-B	interleukin-17B
IL17-C	interleukin-17C
IL-17D	interleukin-17D
IP-10	interferon-gamma-inducible protein 10
MDC	macrophage-derived chemokine
MCP-1	monocyte chemoattractant protein-1
MCP-4	monocyte chemoattractant protein-4
MIP-1α	macrophage inflammatory protein-1α
MIP-1β	macrophage inflammatory protein-1β
PCA	principal component analysis
p-glucose	plasma glucose
PlGF	placental growth factor
SAA	serum amyloid A
Tie-2	tyrosine kinase with immunoglobulin-like loops and epidermal growth factor-like domains 2
TARC	thymus and activation-regulated chemokine
TNF-α	tumour necrosis factor-α
TNF-β	tumour necrosis factor-β
TSLP	thymic stromal lymphopoietin
VEGF-A	vascular endothelial growth factor-A
VEGF-C	vascular endothelial growth factor-C
VEGF-D	vascular endothelial growth factor-D
VEGFR-1/Flt-1	vascular endothelial growth factor receptor-1/Fms-like tyrosine kinase-1
VCAM-1	vascular cell adhesion molecule-1
