# Supplementary material for: Inflammatory and endothelial host responses in community-acquired pneumonia: exploring the relationships with HbA1c, admission plasma glucose, and glycaemic gap—a cross-sectional study
Source: Front Immunol. 2024 May 22;15:1372300. doi: 10.3389/fimmu.2024.1372300 (PMC11150596; doi:10.3389/fimmu.2024.1372300)
Supplement: Supplementary file 1 [file DataSheet_1.docx]

Supplementary Material

Inflammatory and Endothelial Host Responses in Community-Acquired Pneumonia: Exploring the Relationships with HbA1c, Admission Plasma Glucose, and Glycaemic Gap - A Cross-Sectional Study

Arnold Matovu Dungu^1^, Agnete Troen Lundgaard^2^, Camilla Koch Ryrsø^1,3^, Maria Hein Hegelund^1^, Andreas Vestergaard Jensen^1^, Peter Lommer Kristensen^4,7^, Rikke Krogh-Madsen^3,6^, Daniel Faurholt-Jepsen^5,7^, Sisse Rye Ostrowski^7,8^, Karina Banasik^2^, Birgitte Lindegaard^1,3,7^

^1^ Department of Pulmonary and Infectious Diseases, Copenhagen University Hospital, North Zealand, Denmark.

^2^Novo Nordisk Foundation Center for Protein Research, Faculty of Health and Medical Sciences, University of Copenhagen, Copenhagen, Denmark

^3^Centre for Physical Activity Research, Rigshospitalet, University of Copenhagen, Denmark.

^4^Department of Endocrinology and Nephrology, Copenhagen University Hospital, North Zealand, Denmark

^5^Department of Infectious Diseases, Copenhagen University Hospital, Rigshospitalet

^6^Department of Infectious Diseases, Copenhagen University Hospital, Hvidovre, Copenhagen, Denmark

^7^Department of Clinical Medicine, Faculty of Health and Medical Sciences, University of Copenhagen, Copenhagen, Denmark

^8^Department of Clinical Immunology, Copenhagen University Hospital, Rigshospitalet, Copenhagen, Denmark

*** Correspondence:**
Arnold Matovu Dungu

Email address: arnold.matovu.dungu@regionh.dk

# Supplementary methods

## Sample pre-processing

The light signal intensity was used to quantify analyte concentration instead of calculated concentrations to avoid noise introduced by standard curves. For example, 17.7 % of the calculated concentrations were below the lower limit of detection and imputed as half of the “lower limit of detection”, which is a potential source of bias. However, data from 2 unpublished studies (pending publication) showed that using the signal values instead of calculated concentrations does not skew test statistics or introduce bias.

Principal component analysis (PCA), density, and box plots were used panel-wise to assess signal value distribution, extreme outliers, and plate-dependent batch effects. Sample pre-processing of the light signal consisted of logarithmic transformation to achieve a normal distribution, removal of outliers based on visual inspection of principal component analysis (PCA) plots of principal component (PC) 1 and PC2 with self-defined outlier limits, and correction for batch-effects with median normalisation. After sample pre-processing, 14, 20, 10, 56, 30, and 8 measurements were removed from the Angiogenesis panel 1, Chemokine Panel 1, Cytokine Panel 1, Cytokine Panel 2, Pro-inflammatory Panel 1 and Vascular Injury Panel 2, respectively. IL-8 and VEGF-A were measured on 2 different panels. IL-8 measured on the Chemokine Panel 1 and vascular endothelial growth factor-A (VEGF-A) measured on the Angiogenesis Panel 1 were used for the analyses.

## Statistical analysis – missing data

Some patients were expected to be unable to fully participate in all planned investigations due to disease severity or pre-existing conditions, as most pneumonia patients are elderly with impaired functional ability(1). A logistic regression model was fitted with baseline characteristics as predictors and a binary dummy variable for missing BMI as the dependent outcome. The odds ratio for missing BMI was significantly higher for patients with moderate to severe CAP compared to patients with mild CAP according to the CURB-65 score. However, BMI was similar across the CURB-65 groups in the observed data. Thus, the missing data mechanism for BMI was assumed to be missing at random. Missing data were imputed with mean predictive matching using the Multiple Imputations by Chained Equations (MICE) package in R [using the other variables as predictors] to create 50 multiply imputed datasets with 100 iterations per dataset with good convergence from visual inspection.

# Supplementary Tables

| Supplementary Table 1: Overview of the 6 panels measured | | |
| --- | --- | --- |
| Panel | Biomarkers | Site of analysis |
| Pro-inflammatory Panel 1 | Interferon-gamma (IFN-γ), interleukin-1β (IL-1β), IL-2, IL-4, IL-6, IL-8, IL-10, IL-12p70, IL-13, tumor necrosis factor-α (TNF-α) | Department of Clinical Immunology, Copenhagen University Hospital, Rigshospitalet |
| Cytokine Panel 1 | Granulocyte-macrophage colony-stimulating factor (GM-CSF), IL-1α, IL-5, IL-7, IL-12/IL-23p40, IL-15, IL-16, IL-17A, TNF-β, vascular endothelial growth factor-A (VEGF-A) | Department of Clinical Immunology, Copenhagen University Hospital, Rigshospitalet |
| Cytokine Panel 2 | IL-17A/F, IL-17B, IL17-C, IL-17D, interleukin-1 receptor antagonist (IL-1RA), IL-3, IL-9, thymic stromal lymphopoietin (TSLP) | Technical University of Denmark |
| Chemokine Panel 1 | Eotaxin, eotaxin-3, macrophage inflammatory protein-1α (MIP-1α), MIP-1β, thymus and activation-regulated chemokine (TARC), interferon-gamma-inducible protein-10 (IP-10), IL-8, macrophage-derived chemokine (MDC), monocyte chemoattractant protein-1 (MCP-1), MCP-4 | Technical University of Denmark |
| Angiogenesis Panel 1 | Basic fibroblast growth factor (bFGF), soluble fms-like tyrosine kinase-1 (sFlt-1), placental growth factor (PlGF), soluble tyrosine kinase with immunoglobulin-like loops and epidermal growth factor-like domains-2 (sTie-2), VEGF-A, VEGF-C, VEGF-D | Department of Clinical Immunology, Aarhus University Hospital |
| Vascular Injury Panel 2 | Serum amyloid A (SAA), c-reactive protein (CRP), soluble vascular cell adhesion molecule-1 (sVCAM-1), soluble intercellular adhesion molecule-1 (sICAM-1) | Technical University of Denmark |

| Supplementary Table 2: Pro-inflammatory biomarkers | | |
| --- | --- | --- |
| Biomarker | Main known function^1^ | Cellular sources^1^ |
| CRP | Acute phase response, activates the complement system, opsonisation | Produced in the liver |
| SAA | Acute phase response, chemotaxis, cytokine induction | Produced in the liver |
| TNF-α | Pro-inflammatory, cell death regulation, immune cell activation | Macrophages, T cells, NK cells |
| TNF-β | Pro-inflammatory, cytotoxic, and immunoregulatory activities | T cells, NK cells |
| IL-1α | Pro-inflammatory cytokine, fever induction, and acute phase response | Monocytes, macrophages |
| IL-1β | Pro-inflammatory cytokine, fever induction, and acute phase response | Monocytes, macrophages |
| IL-1RA | Inhibits IL-1α and IL-1β signaling, anti-inflammatory | Monocytes, macrophages |
| IL-3 | Hematopoietic cell growth, differentiation, and survival | T cells, mast cells |
| IL-6 | Pro-inflammatory, acute phase response, B cell differentiation | T cells, macrophages, endothelial cells |
| IL-12/IL-23p40 | Subunit shared by IL-12 and IL-23; involved in T cell differentiation and activation | Dendritic cells, macrophages |
| IL-12p70 | Promotes Th1 cell differentiation, IFN-γ production, and cell-mediated immunity | Dendritic cells, macrophages |
| IL-15 | T cell and NK cell activation, proliferation, and survival | Monocytes, macrophages, dendritic cells |
| ^1^Created with information from these references (2–7) | | |

| Supplementary Table 3: T-cell-derived biomarkers | | |
| --- | --- | --- |
| Biomarker | Main known function^1^ | Cellular sources^1^ |
| IFN-γ | Antiviral, immunoregulatory, and anti-tumor properties | T cells, NK cells |
| IL-2 | T cell proliferation, activation, and differentiation | T cells, dendritic cells |
| IL-4 | Th2 cell differentiation, B cell activation, and antibody class-switching | T cells, mast cells |
| IL-5 | Eosinophil activation and differentiation, B cell growth and differentiation | T cells, mast cells |
| IL-9 | T cell growth factor, mast cell and hematopoietic precursor growth | T cells |
| IL-10 | Anti-inflammatory, downregulates Th1 cytokine production | T cells, B cells, macrophages |
| IL-13 | Th2 cytokine, B cell proliferation, and immunoglobulin production | T cells, NK cells, mast cells |
| IL-16 | Chemotactic factor for CD4+ T cells, monocytes, and eosinophils | T cells, epithelial cells |
| IL-17A | Pro-inflammatory, involved in autoimmune and inflammatory responses | Th17 cells, CD8+ T cells, NK cells |
| IL-17A/F | Pro-inflammatory, involved in autoimmune and inflammatory responses | Th17 cells |
| IL-17B | Role in inflammation and autoimmune diseases | T cells, B cells, neutrophils |
| IL-17C | Involved in mucosal host defence and pro-inflammatory responses | Epithelial cells, immune cells |
| IL-17D | Role in immune responses, particularly in the context of viral infections | Endothelial cells, monocytes, NK cells |
| ^1^Created with information from these references (2–7) | | |

| Supplementary Table 4: Chemokines | | |
| --- | --- | --- |
| Biomarker | Main known function^1^ | Cellular sources^1^ |
| eotaxin | Chemotaxis of eosinophils, basophils, and Th2 lymphocytes | Endothelial cells, fibroblasts, epithelial cells |
| eotaxin-3 | Chemotaxis of eosinophils, basophils, and Th2 lymphocytes | Endothelial cells, fibroblasts, epithelial cells |
| MIP-1α | Chemotaxis and activation of monocytes, macrophages, and granulocytes | Monocytes, macrophages, T cells, dendritic cells |
| MIP-1β | Chemotaxis and activation of monocytes, macrophages, and granulocytes | Monocytes, macrophages, T cells, dendritic cells |
| TARC | Chemotaxis of Th2 lymphocytes | Dendritic cells, endothelial cells, fibroblasts |
| IP-10 | Chemotaxis of monocytes, T cells, NK cells, and dendritic cells | Monocytes, endothelial cells, fibroblasts |
| IL-8 | Neutrophil chemotaxis and activation, angiogenesis | Macrophages, endothelial cells, epithelial cells |
| MDC | Chemotaxis of monocytes, dendritic cells, and NK cells | Dendritic cells, macrophages, monocytes |
| MCP-1 | Chemotaxis and activation of monocytes, memory T cells, and basophils | Monocytes, endothelial cells, fibroblasts |
| MCP-4 | Chemotaxis of eosinophils, basophils, and Th2 lymphocytes | Endothelial cells, fibroblasts, epithelial cells |
| ^1^Created with information from these references (2–7) | | |

| Supplementary Table 5: endothelial biomarkers and growth factors | | |
| --- | --- | --- |
| Biomarker | Main known function^1^ | Cellular sources^1^ |
| bFGF | Angiogenesis, wound healing, tissue repair | Endothelial cells, fibroblasts, macrophages |
| Flt-1 | VEGF receptor, regulates angiogenesis, vascular development | Endothelial cells |
| Tie-2 | Angiopoietin receptor, regulates angiogenesis, vascular stability | Endothelial cells |
| IL-7 | Lymphopoiesis, T and B cell survival and development | Stromal cells, epithelial cells |
| PlGF | Angiogenesis, monocyte activation | Endothelial cells, trophoblasts |
| VEGF-A | Angiogenesis, endothelial cell growth, vascular permeability | Endothelial cells, macrophages |
| VEGF-C | Lymphangiogenesis, angiogenesis | Endothelial cells, macrophages |
| VEGF-D | Lymphangiogenesis, angiogenesis | Endothelial cells, macrophages |
| TSLP | Th2 immune response, pro-allergic response | Epithelial cells, fibroblasts, mast cells |
| GM-CSF | Granulocyte and macrophage development, immune activation | T cells, macrophages, endothelial cells |
| sICAM-1 | Cell adhesion, leukocyte trafficking, inflammatory response | Endothelial cells, immune cells |
| sVCAM-1 | Cell adhesion, leukocyte trafficking, inflammatory response | Endothelial cells, immune cells |
| ^1^Created with information from these references (2–7) | | |

| Supplementary Table 6: Base model summaries with diabetes status as predictor  Euglycaemia (no diabetes and HbA1c < 39 mmol/mol) was reference for each comparison | | | | | | |
| --- | --- | --- | --- | --- | --- | --- |
| Analyte | Predictor | Estimate | 95% CI lower | 95% CI higher | P-value | FDR-adjusted p-value |
| CRP | Known diabetes | 0.94 | 0.81 | 1.10 | 0.466 | 0.957 |
|  | Prediabetes | 0.99 | 0.88 | 1.13 | 0.910 | 0.980 |
|  | Unknown diabetes | 1.10 | 0.82 | 1.48 | 0.509 | 0.967 |
| Eotaxin | Known diabetes | 1.24 | 1.06 | 1.45 | 0.006 | 0.316 |
|  | Prediabetes | 0.96 | 0.85 | 1.10 | 0.579 | 0.980 |
|  | Unknown diabetes | 1.01 | 0.75 | 1.36 | 0.937 | 0.980 |
| Eotaxin-3 | Known diabetes | 1.16 | 0.94 | 1.42 | 0.164 | 0.742 |
|  | Prediabetes | 1.04 | 0.88 | 1.23 | 0.626 | 0.980 |
|  | Unknown diabetes | 1.52 | 1.03 | 2.26 | 0.037 | 0.386 |
| Flt-1 | Known diabetes | 1.09 | 0.96 | 1.23 | 0.189 | 0.791 |
|  | Prediabetes | 1.09 | 0.98 | 1.21 | 0.109 | 0.609 |
|  | Unknown diabetes | 1.37 | 1.07 | 1.74 | 0.011 | 0.316 |
| GM-CSF | Known diabetes | 1.01 | 0.96 | 1.07 | 0.671 | 0.980 |
|  | Prediabetes | 1.00 | 0.96 | 1.05 | 0.934 | 0.980 |
|  | Unknown diabetes | 1.03 | 0.93 | 1.15 | 0.575 | 0.980 |
| IFN-γ | Known diabetes | 1.25 | 0.86 | 1.81 | 0.244 | 0.867 |
|  | Prediabetes | 1.35 | 1.00 | 1.83 | 0.051 | 0.443 |
|  | Unknown diabetes | 1.11 | 0.55 | 2.24 | 0.781 | 0.980 |
| IL-10 | Known diabetes | 0.90 | 0.72 | 1.12 | 0.328 | 0.911 |
|  | Prediabetes | 0.89 | 0.74 | 1.06 | 0.194 | 0.805 |
|  | Unknown diabetes | 0.99 | 0.65 | 1.51 | 0.961 | 0.984 |
| IL-12 | Known diabetes | 1.18 | 0.95 | 1.47 | 0.141 | 0.691 |
|  | Prediabetes | 0.79 | 0.66 | 0.95 | 0.011 | 0.316 |
|  | Unknown diabetes | 0.96 | 0.63 | 1.47 | 0.858 | 0.980 |
| IL-12p70 | Known diabetes | 0.98 | 0.91 | 1.06 | 0.691 | 0.980 |
|  | Prediabetes | 0.97 | 0.91 | 1.03 | 0.292 | 0.911 |
|  | Unknown diabetes | 0.86 | 0.74 | 1.00 | 0.046 | 0.427 |
| IL-13 | Known diabetes | 0.96 | 0.89 | 1.03 | 0.265 | 0.895 |
|  | Prediabetes | 1.00 | 0.94 | 1.06 | 0.871 | 0.980 |
|  | Unknown diabetes | 0.97 | 0.84 | 1.11 | 0.624 | 0.980 |
| IL-15 | Known diabetes | 1.20 | 1.07 | 1.33 | 0.001 | 0.308 |
|  | Prediabetes | 1.15 | 1.05 | 1.26 | 0.002 | 0.308 |
|  | Unknown diabetes | 1.34 | 1.09 | 1.65 | 0.005 | 0.316 |
| IL-16 | Known diabetes | 1.17 | 1.02 | 1.34 | 0.027 | 0.336 |
|  | Prediabetes | 1.08 | 0.96 | 1.20 | 0.184 | 0.780 |
|  | Unknown diabetes | 1.17 | 0.90 | 1.51 | 0.242 | 0.865 |
| IL-17A | Known diabetes | 1.25 | 1.04 | 1.51 | 0.019 | 0.335 |
|  | Prediabetes | 0.95 | 0.82 | 1.11 | 0.517 | 0.969 |
|  | Unknown diabetes | 1.09 | 0.76 | 1.56 | 0.632 | 0.980 |
| IL-17A/F | Known diabetes | 1.07 | 0.98 | 1.18 | 0.135 | 0.688 |
|  | Prediabetes | 1.05 | 0.97 | 1.13 | 0.220 | 0.819 |
|  | Unknown diabetes | 0.99 | 0.83 | 1.18 | 0.949 | 0.980 |
| IL-17B | Known diabetes | 1.03 | 0.93 | 1.13 | 0.601 | 0.980 |
|  | Prediabetes | 0.98 | 0.91 | 1.07 | 0.680 | 0.980 |
|  | Unknown diabetes | 1.01 | 0.84 | 1.22 | 0.911 | 0.980 |
| IL-17C | Known diabetes | 1.11 | 1.01 | 1.22 | 0.024 | 0.335 |
|  | Prediabetes | 0.99 | 0.92 | 1.07 | 0.782 | 0.980 |
|  | Unknown diabetes | 0.98 | 0.82 | 1.17 | 0.839 | 0.980 |
| IL-17D | Known diabetes | 1.02 | 0.94 | 1.11 | 0.594 | 0.980 |
|  | Prediabetes | 1.04 | 0.98 | 1.11 | 0.201 | 0.808 |
|  | Unknown diabetes | 0.94 | 0.81 | 1.10 | 0.449 | 0.957 |
| IL-1RA | Known diabetes | 1.31 | 0.95 | 1.79 | 0.096 | 0.582 |
|  | Prediabetes | 1.08 | 0.83 | 1.39 | 0.575 | 0.980 |
|  | Unknown diabetes | 1.52 | 0.84 | 2.77 | 0.168 | 0.744 |
| IL-1α | Known diabetes | 0.90 | 0.81 | 1.01 | 0.068 | 0.506 |
|  | Prediabetes | 0.98 | 0.90 | 1.07 | 0.656 | 0.980 |
|  | Unknown diabetes | 1.05 | 0.85 | 1.29 | 0.671 | 0.980 |
| IL-1β | Known diabetes | 0.99 | 0.91 | 1.07 | 0.771 | 0.980 |
|  | Prediabetes | 1.06 | 0.99 | 1.13 | 0.108 | 0.605 |
|  | Unknown diabetes | 1.00 | 0.86 | 1.17 | 0.981 | 0.995 |
| IL-2 | Known diabetes | 1.00 | 0.89 | 1.11 | 0.935 | 0.980 |
|  | Prediabetes | 0.99 | 0.91 | 1.08 | 0.835 | 0.980 |
|  | Unknown diabetes | 1.01 | 0.82 | 1.24 | 0.933 | 0.980 |
| IL-3 | Known diabetes | 0.97 | 0.86 | 1.09 | 0.616 | 0.980 |
|  | Prediabetes | 1.12 | 1.02 | 1.24 | 0.015 | 0.335 |
|  | Unknown diabetes | 1.00 | 0.81 | 1.25 | 0.970 | 0.989 |
| IL-4 | Known diabetes | 1.00 | 0.91 | 1.11 | 0.996 | 0.999 |
|  | Prediabetes | 1.02 | 0.94 | 1.10 | 0.685 | 0.980 |
|  | Unknown diabetes | 0.91 | 0.76 | 1.10 | 0.353 | 0.911 |
| IL-5 | Known diabetes | 0.99 | 0.82 | 1.20 | 0.895 | 0.980 |
|  | Prediabetes | 0.95 | 0.81 | 1.11 | 0.545 | 0.980 |
|  | Unknown diabetes | 1.05 | 0.73 | 1.51 | 0.807 | 0.980 |
| IL-6 | Known diabetes | 1.03 | 0.72 | 1.48 | 0.852 | 0.980 |
|  | Prediabetes | 1.02 | 0.76 | 1.37 | 0.889 | 0.980 |
|  | Unknown diabetes | 1.05 | 0.53 | 2.06 | 0.890 | 0.980 |
| IL-7 | Known diabetes | 1.01 | 0.86 | 1.18 | 0.927 | 0.980 |
|  | Prediabetes | 0.97 | 0.85 | 1.10 | 0.627 | 0.980 |
|  | Unknown diabetes | 1.15 | 0.85 | 1.55 | 0.357 | 0.911 |
| IL-8 | Known diabetes | 1.09 | 1.01 | 1.17 | 0.036 | 0.383 |
|  | Prediabetes | 1.02 | 0.96 | 1.09 | 0.534 | 0.980 |
|  | Unknown diabetes | 1.12 | 0.97 | 1.30 | 0.125 | 0.650 |
| IL-9 | Known diabetes | 1.13 | 0.99 | 1.29 | 0.080 | 0.535 |
|  | Prediabetes | 1.14 | 1.02 | 1.28 | 0.017 | 0.335 |
|  | Unknown diabetes | 1.15 | 0.89 | 1.49 | 0.279 | 0.907 |
| IP-10 | Known diabetes | 1.05 | 0.79 | 1.39 | 0.743 | 0.980 |
|  | Prediabetes | 1.12 | 0.88 | 1.41 | 0.356 | 0.911 |
|  | Unknown diabetes | 1.37 | 0.80 | 2.35 | 0.250 | 0.868 |
| MCP-1 | Known diabetes | 1.07 | 0.88 | 1.29 | 0.504 | 0.967 |
|  | Prediabetes | 0.97 | 0.83 | 1.12 | 0.650 | 0.980 |
|  | Unknown diabetes | 0.90 | 0.63 | 1.28 | 0.543 | 0.980 |
| MCP-4 | Known diabetes | 1.15 | 0.98 | 1.36 | 0.085 | 0.549 |
|  | Prediabetes | 0.96 | 0.84 | 1.10 | 0.558 | 0.980 |
|  | Unknown diabetes | 1.13 | 0.83 | 1.55 | 0.435 | 0.957 |
| MDC | Known diabetes | 0.98 | 0.84 | 1.15 | 0.828 | 0.980 |
|  | Prediabetes | 0.92 | 0.81 | 1.04 | 0.195 | 0.806 |
|  | Unknown diabetes | 1.02 | 0.76 | 1.38 | 0.876 | 0.980 |
| MIP-1α | Known diabetes | 0.99 | 0.87 | 1.13 | 0.916 | 0.980 |
|  | Prediabetes | 1.02 | 0.91 | 1.14 | 0.713 | 0.980 |
|  | Unknown diabetes | 1.12 | 0.87 | 1.44 | 0.389 | 0.927 |
| MIP-1β | Known diabetes | 0.96 | 0.76 | 1.20 | 0.692 | 0.980 |
|  | Prediabetes | 0.88 | 0.73 | 1.06 | 0.170 | 0.749 |
|  | Unknown diabetes | 1.23 | 0.80 | 1.89 | 0.337 | 0.911 |
| PlGF | Known diabetes | 0.97 | 0.89 | 1.05 | 0.478 | 0.960 |
|  | Prediabetes | 0.99 | 0.93 | 1.06 | 0.856 | 0.980 |
|  | Unknown diabetes | 1.08 | 0.92 | 1.26 | 0.354 | 0.911 |
| SAA | Known diabetes | 0.98 | 0.81 | 1.19 | 0.824 | 0.980 |
|  | Prediabetes | 1.07 | 0.91 | 1.25 | 0.426 | 0.956 |
|  | Unknown diabetes | 1.20 | 0.83 | 1.74 | 0.327 | 0.911 |
| TARC | Known diabetes | 1.12 | 0.90 | 1.40 | 0.299 | 0.911 |
|  | Prediabetes | 0.97 | 0.81 | 1.17 | 0.765 | 0.980 |
|  | Unknown diabetes | 0.96 | 0.63 | 1.46 | 0.837 | 0.980 |
| TLSP | Known diabetes | 1.12 | 0.96 | 1.31 | 0.165 | 0.742 |
|  | Prediabetes | 1.11 | 0.98 | 1.26 | 0.115 | 0.634 |
|  | Unknown diabetes | 1.12 | 0.83 | 1.51 | 0.444 | 0.957 |
| TNF-α | Known diabetes | 1.04 | 0.90 | 1.20 | 0.612 | 0.980 |
|  | Prediabetes | 0.98 | 0.87 | 1.11 | 0.798 | 0.980 |
|  | Unknown diabetes | 1.07 | 0.81 | 1.41 | 0.649 | 0.980 |
| TNF-β | Known diabetes | 1.03 | 0.98 | 1.08 | 0.311 | 0.911 |
|  | Prediabetes | 0.98 | 0.94 | 1.02 | 0.337 | 0.911 |
|  | Unknown diabetes | 0.97 | 0.89 | 1.07 | 0.580 | 0.980 |
| Tie-2 | Known diabetes | 1.09 | 1.02 | 1.17 | 0.012 | 0.316 |
|  | Prediabetes | 1.00 | 0.95 | 1.06 | 0.905 | 0.980 |
|  | Unknown diabetes | 0.99 | 0.87 | 1.13 | 0.860 | 0.980 |
| VEGF-A | Known diabetes | 1.01 | 0.80 | 1.27 | 0.931 | 0.980 |
|  | Prediabetes | 1.01 | 0.84 | 1.23 | 0.884 | 0.980 |
|  | Unknown diabetes | 1.36 | 0.87 | 2.11 | 0.172 | 0.750 |
| VEGF-C | Known diabetes | 1.06 | 0.95 | 1.17 | 0.298 | 0.911 |
|  | Prediabetes | 0.98 | 0.91 | 1.07 | 0.715 | 0.980 |
|  | Unknown diabetes | 1.07 | 0.88 | 1.29 | 0.507 | 0.967 |
| VEGF-D | Known diabetes | 1.15 | 1.02 | 1.30 | 0.023 | 0.335 |
|  | Prediabetes | 0.97 | 0.88 | 1.07 | 0.556 | 0.980 |
|  | Unknown diabetes | 1.09 | 0.87 | 1.37 | 0.451 | 0.957 |
| bFGF | Known diabetes | 1.27 | 1.00 | 1.61 | 0.049 | 0.443 |
|  | Prediabetes | 0.98 | 0.80 | 1.19 | 0.818 | 0.980 |
|  | Unknown diabetes | 1.24 | 0.79 | 1.95 | 0.351 | 0.911 |
| sICAM-1 | Known diabetes | 1.00 | 0.91 | 1.09 | 0.948 | 0.980 |
|  | Prediabetes | 1.00 | 0.92 | 1.07 | 0.910 | 0.980 |
|  | Unknown diabetes | 0.97 | 0.82 | 1.15 | 0.743 | 0.980 |
| sVCAM-1 | Known diabetes | 1.13 | 1.03 | 1.23 | 0.007 | 0.316 |
|  | Prediabetes | 0.98 | 0.92 | 1.06 | 0.654 | 0.980 |
|  | Unknown diabetes | 1.00 | 0.84 | 1.18 | 0.986 | 0.995 |
| Abbreviations: CI, confidence interval, FDR, false discovery rate | | | | | | |

| Supplementary Table 7: Summaries of adjusted models with diabetes status as the predictor  Euglycaemia (no diabetes and HbA1c < 39 mmol/mol) was the reference for each comparison | | | | | | |
| --- | --- | --- | --- | --- | --- | --- |
| Analyte | Predictor | Estimate | 95% CI lower | 95% CI higher | P-value | FDR-adjusted p-value |
| CRP | Known diabetes | 1.03 | 0.87 | 1.23 | 0.713 | 0.980 |
|  | Prediabetes | 1.01 | 0.88 | 1.16 | 0.871 | 0.980 |
|  | Unknown diabetes | 1.10 | 0.78 | 1.53 | 0.591 | 0.980 |
| Eotaxin | Known diabetes | 1.16 | 0.97 | 1.40 | 0.107 | 0.605 |
|  | Prediabetes | 0.97 | 0.84 | 1.12 | 0.665 | 0.980 |
|  | Unknown diabetes | 0.93 | 0.66 | 1.32 | 0.677 | 0.980 |
| Eotaxin-3 | Known diabetes | 1.11 | 0.87 | 1.41 | 0.391 | 0.927 |
|  | Prediabetes | 0.97 | 0.80 | 1.17 | 0.726 | 0.980 |
|  | Unknown diabetes | 1.07 | 0.68 | 1.68 | 0.768 | 0.980 |
| Flt-1 | Known diabetes | 1.07 | 0.93 | 1.24 | 0.341 | 0.911 |
|  | Prediabetes | 1.05 | 0.94 | 1.17 | 0.421 | 0.949 |
|  | Unknown diabetes | 1.02 | 0.78 | 1.34 | 0.894 | 0.980 |
| GM-CSF | Known diabetes | 1.04 | 0.98 | 1.11 | 0.224 | 0.831 |
|  | Prediabetes | 1.00 | 0.95 | 1.05 | 0.960 | 0.984 |
|  | Unknown diabetes | 1.02 | 0.91 | 1.16 | 0.696 | 0.980 |
| IFN-γ | Known diabetes | 1.21 | 0.81 | 1.82 | 0.352 | 0.911 |
|  | Prediabetes | 1.39 | 1.01 | 1.91 | 0.044 | 0.410 |
|  | Unknown diabetes | 1.29 | 0.60 | 2.76 | 0.516 | 0.969 |
| IL-10 | Known diabetes | 0.99 | 0.77 | 1.28 | 0.950 | 0.980 |
|  | Prediabetes | 0.85 | 0.69 | 1.03 | 0.104 | 0.604 |
|  | Unknown diabetes | 0.89 | 0.55 | 1.43 | 0.625 | 0.980 |
| IL-12 | Known diabetes | 1.12 | 0.88 | 1.43 | 0.361 | 0.911 |
|  | Prediabetes | 0.79 | 0.65 | 0.96 | 0.018 | 0.335 |
|  | Unknown diabetes | 0.97 | 0.61 | 1.55 | 0.911 | 0.980 |
| IL-12p70 | Known diabetes | 0.95 | 0.87 | 1.03 | 0.209 | 0.811 |
|  | Prediabetes | 0.96 | 0.90 | 1.03 | 0.258 | 0.875 |
|  | Unknown diabetes | 0.83 | 0.71 | 0.98 | 0.025 | 0.335 |
| IL-13 | Known diabetes | 0.94 | 0.86 | 1.02 | 0.163 | 0.742 |
|  | Prediabetes | 0.97 | 0.91 | 1.04 | 0.387 | 0.927 |
|  | Unknown diabetes | 0.95 | 0.81 | 1.12 | 0.550 | 0.980 |
| IL-15 | Known diabetes | 1.21 | 1.07 | 1.38 | 0.003 | 0.308 |
|  | Prediabetes | 1.13 | 1.02 | 1.24 | 0.019 | 0.335 |
|  | Unknown diabetes | 1.35 | 1.07 | 1.72 | 0.013 | 0.316 |
| IL-16 | Known diabetes | 1.14 | 0.97 | 1.34 | 0.104 | 0.604 |
|  | Prediabetes | 1.05 | 0.93 | 1.20 | 0.409 | 0.942 |
|  | Unknown diabetes | 1.03 | 0.76 | 1.38 | 0.870 | 0.980 |
| IL-17A | Known diabetes | 1.27 | 1.03 | 1.55 | 0.023 | 0.335 |
|  | Prediabetes | 0.94 | 0.80 | 1.10 | 0.458 | 0.957 |
|  | Unknown diabetes | 0.96 | 0.65 | 1.40 | 0.818 | 0.980 |
| IL-17A/F | Known diabetes | 1.10 | 0.98 | 1.23 | 0.104 | 0.604 |
|  | Prediabetes | 1.05 | 0.96 | 1.15 | 0.285 | 0.911 |
|  | Unknown diabetes | 0.96 | 0.78 | 1.19 | 0.728 | 0.980 |
| IL-17B | Known diabetes | 0.98 | 0.88 | 1.08 | 0.647 | 0.980 |
|  | Prediabetes | 0.98 | 0.90 | 1.06 | 0.560 | 0.980 |
|  | Unknown diabetes | 1.00 | 0.82 | 1.21 | 0.984 | 0.995 |
| IL-17C | Known diabetes | 1.12 | 1.00 | 1.26 | 0.043 | 0.407 |
|  | Prediabetes | 0.99 | 0.91 | 1.08 | 0.814 | 0.980 |
|  | Unknown diabetes | 0.89 | 0.72 | 1.10 | 0.293 | 0.911 |
| IL-17D | Known diabetes | 1.03 | 0.93 | 1.13 | 0.583 | 0.980 |
|  | Prediabetes | 1.04 | 0.97 | 1.12 | 0.292 | 0.911 |
|  | Unknown diabetes | 0.95 | 0.79 | 1.13 | 0.543 | 0.980 |
| IL-1RA | Known diabetes | 1.21 | 0.86 | 1.69 | 0.278 | 0.907 |
|  | Prediabetes | 1.00 | 0.77 | 1.30 | 0.991 | 0.995 |
|  | Unknown diabetes | 1.33 | 0.71 | 2.51 | 0.377 | 0.918 |
| IL-1α | Known diabetes | 0.89 | 0.78 | 1.01 | 0.067 | 0.506 |
|  | Prediabetes | 0.98 | 0.88 | 1.08 | 0.643 | 0.980 |
|  | Unknown diabetes | 1.00 | 0.78 | 1.28 | 0.989 | 0.995 |
| IL-1β | Known diabetes | 1.02 | 0.92 | 1.12 | 0.756 | 0.980 |
|  | Prediabetes | 1.03 | 0.96 | 1.12 | 0.370 | 0.915 |
|  | Unknown diabetes | 0.99 | 0.82 | 1.18 | 0.870 | 0.980 |
| IL-2 | Known diabetes | 0.99 | 0.87 | 1.12 | 0.860 | 0.980 |
|  | Prediabetes | 0.99 | 0.90 | 1.09 | 0.842 | 0.980 |
|  | Unknown diabetes | 0.95 | 0.76 | 1.21 | 0.699 | 0.980 |
| IL-3 | Known diabetes | 0.96 | 0.84 | 1.11 | 0.612 | 0.980 |
|  | Prediabetes | 1.12 | 1.00 | 1.26 | 0.041 | 0.404 |
|  | Unknown diabetes | 1.01 | 0.77 | 1.32 | 0.952 | 0.980 |
| IL-4 | Known diabetes | 0.97 | 0.88 | 1.08 | 0.633 | 0.980 |
|  | Prediabetes | 1.00 | 0.92 | 1.09 | 0.942 | 0.980 |
|  | Unknown diabetes | 0.92 | 0.76 | 1.12 | 0.403 | 0.936 |
| IL-5 | Known diabetes | 1.14 | 0.91 | 1.42 | 0.254 | 0.870 |
|  | Prediabetes | 1.05 | 0.88 | 1.25 | 0.614 | 0.980 |
|  | Unknown diabetes | 1.03 | 0.68 | 1.56 | 0.900 | 0.980 |
| IL-6 | Known diabetes | 1.22 | 0.84 | 1.77 | 0.288 | 0.911 |
|  | Prediabetes | 1.06 | 0.79 | 1.41 | 0.715 | 0.980 |
|  | Unknown diabetes | 0.94 | 0.47 | 1.88 | 0.858 | 0.980 |
| IL-7 | Known diabetes | 1.05 | 0.88 | 1.25 | 0.584 | 0.980 |
|  | Prediabetes | 0.96 | 0.84 | 1.10 | 0.585 | 0.980 |
|  | Unknown diabetes | 1.36 | 0.98 | 1.89 | 0.066 | 0.505 |
| IL-8 | Known diabetes | 1.04 | 0.96 | 1.14 | 0.331 | 0.911 |
|  | Prediabetes | 1.00 | 0.94 | 1.08 | 0.887 | 0.980 |
|  | Unknown diabetes | 0.97 | 0.82 | 1.14 | 0.720 | 0.980 |
| IL-9 | Known diabetes | 1.17 | 0.99 | 1.38 | 0.066 | 0.505 |
|  | Prediabetes | 1.13 | 0.99 | 1.29 | 0.060 | 0.476 |
|  | Unknown diabetes | 1.14 | 0.84 | 1.57 | 0.399 | 0.936 |
| IP-10 | Known diabetes | 1.03 | 0.75 | 1.41 | 0.869 | 0.980 |
|  | Prediabetes | 1.09 | 0.84 | 1.40 | 0.519 | 0.969 |
|  | Unknown diabetes | 1.36 | 0.74 | 2.49 | 0.316 | 0.911 |
| MCP-1 | Known diabetes | 1.03 | 0.83 | 1.27 | 0.779 | 0.980 |
|  | Prediabetes | 0.94 | 0.80 | 1.11 | 0.479 | 0.960 |
|  | Unknown diabetes | 0.75 | 0.50 | 1.12 | 0.159 | 0.742 |
| MCP-4 | Known diabetes | 1.09 | 0.90 | 1.33 | 0.360 | 0.911 |
|  | Prediabetes | 0.97 | 0.83 | 1.13 | 0.680 | 0.980 |
|  | Unknown diabetes | 1.14 | 0.79 | 1.65 | 0.469 | 0.957 |
| MDC | Known diabetes | 0.91 | 0.76 | 1.09 | 0.295 | 0.911 |
|  | Prediabetes | 0.91 | 0.79 | 1.05 | 0.185 | 0.781 |
|  | Unknown diabetes | 1.12 | 0.80 | 1.56 | 0.519 | 0.969 |
| MIP-1α | Known diabetes | 0.98 | 0.85 | 1.13 | 0.750 | 0.980 |
|  | Prediabetes | 1.00 | 0.90 | 1.12 | 0.946 | 0.980 |
|  | Unknown diabetes | 1.06 | 0.81 | 1.39 | 0.660 | 0.980 |
| MIP-1β | Known diabetes | 1.05 | 0.83 | 1.32 | 0.701 | 0.980 |
|  | Prediabetes | 0.89 | 0.74 | 1.07 | 0.203 | 0.808 |
|  | Unknown diabetes | 1.09 | 0.70 | 1.69 | 0.698 | 0.980 |
| PlGF | Known diabetes | 0.98 | 0.90 | 1.08 | 0.742 | 0.980 |
|  | Prediabetes | 1.00 | 0.93 | 1.08 | 0.897 | 0.980 |
|  | Unknown diabetes | 1.04 | 0.87 | 1.24 | 0.654 | 0.980 |
| SAA | Known diabetes | 1.10 | 0.88 | 1.37 | 0.389 | 0.927 |
|  | Prediabetes | 1.08 | 0.91 | 1.28 | 0.390 | 0.927 |
|  | Unknown diabetes | 1.14 | 0.76 | 1.73 | 0.529 | 0.980 |
| TARC | Known diabetes | 1.09 | 0.83 | 1.41 | 0.541 | 0.980 |
|  | Prediabetes | 0.97 | 0.79 | 1.20 | 0.798 | 0.980 |
|  | Unknown diabetes | 1.03 | 0.63 | 1.70 | 0.893 | 0.980 |
| TLSP | Known diabetes | 1.13 | 0.95 | 1.35 | 0.176 | 0.752 |
|  | Prediabetes | 1.10 | 0.96 | 1.27 | 0.176 | 0.752 |
|  | Unknown diabetes | 1.03 | 0.74 | 1.44 | 0.852 | 0.980 |
| TNF-α | Known diabetes | 1.03 | 0.88 | 1.20 | 0.707 | 0.980 |
|  | Prediabetes | 0.96 | 0.85 | 1.08 | 0.461 | 0.957 |
|  | Unknown diabetes | 0.98 | 0.74 | 1.30 | 0.877 | 0.980 |
| TNF-β | Known diabetes | 1.02 | 0.96 | 1.08 | 0.517 | 0.969 |
|  | Prediabetes | 0.99 | 0.95 | 1.04 | 0.689 | 0.980 |
|  | Unknown diabetes | 0.97 | 0.87 | 1.08 | 0.572 | 0.980 |
| Tie-2 | Known diabetes | 1.11 | 1.02 | 1.21 | 0.011 | 0.316 |
|  | Prediabetes | 1.01 | 0.94 | 1.08 | 0.824 | 0.980 |
|  | Unknown diabetes | 1.00 | 0.86 | 1.17 | 0.999 | 0.999 |
| VEGF-A | Known diabetes | 1.21 | 0.94 | 1.57 | 0.139 | 0.691 |
|  | Prediabetes | 1.05 | 0.86 | 1.29 | 0.610 | 0.980 |
|  | Unknown diabetes | 1.51 | 0.93 | 2.45 | 0.099 | 0.592 |
| VEGF-C | Known diabetes | 1.05 | 0.93 | 1.18 | 0.418 | 0.949 |
|  | Prediabetes | 0.97 | 0.89 | 1.07 | 0.588 | 0.980 |
|  | Unknown diabetes | 1.09 | 0.87 | 1.36 | 0.458 | 0.957 |
| VEGF-D | Known diabetes | 1.20 | 1.05 | 1.38 | 0.008 | 0.316 |
|  | Prediabetes | 0.94 | 0.84 | 1.04 | 0.238 | 0.865 |
|  | Unknown diabetes | 1.01 | 0.78 | 1.30 | 0.967 | 0.987 |
| bFGF | Known diabetes | 1.36 | 1.04 | 1.78 | 0.027 | 0.336 |
|  | Prediabetes | 0.98 | 0.79 | 1.22 | 0.864 | 0.980 |
|  | Unknown diabetes | 1.33 | 0.79 | 2.22 | 0.278 | 0.907 |
| sICAM-1 | Known diabetes | 0.96 | 0.86 | 1.06 | 0.390 | 0.927 |
|  | Prediabetes | 0.97 | 0.89 | 1.05 | 0.409 | 0.942 |
|  | Unknown diabetes | 0.96 | 0.79 | 1.17 | 0.713 | 0.980 |
| sVCAM-1 | Known diabetes | 1.11 | 1.00 | 1.23 | 0.040 | 0.404 |
|  | Prediabetes | 0.97 | 0.89 | 1.05 | 0.453 | 0.957 |
|  | Unknown diabetes | 0.89 | 0.73 | 1.07 | 0.213 | 0.811 |
| Abbreviations: CI, confidence interval, FDR, false discovery rate | | | | | | |

| Supplementary Table 8: Summaries of base models with acute hyperglycaemia as the predictor  Euglycaemia (admission p-glucose < 6.0 mmol/L) was the reference for each comparison | | | | | | |
| --- | --- | --- | --- | --- | --- | --- |
| Analyte | Predictor | Estimate | 95% CI lower | 95% CI higher | P-value | FDR-adjusted p-value |
| CRP | Mild (p-glucose ≥ 6.0 and <11 mmol/L) | 1.17 | 1.01 | 1.35 | 0.032 | 0.376 |
|  | Severe (p-glucose ≥ 11.0 mmol/L) | 1.10 | 0.90 | 1.35 | 0.362 | 0.911 |
| Eotaxin | Mild (p-glucose ≥ 6.0 and <11 mmol/L) | 1.01 | 0.87 | 1.17 | 0.914 | 0.980 |
|  | Severe (p-glucose ≥ 11.0 mmol/L) | 1.19 | 0.96 | 1.47 | 0.107 | 0.605 |
| Eotaxin-3 | Mild (p-glucose ≥ 6.0 and <11 mmol/L) | 1.14 | 0.93 | 1.38 | 0.198 | 0.806 |
|  | Severe (p-glucose ≥ 11.0 mmol/L) | 1.08 | 0.82 | 1.43 | 0.591 | 0.980 |
| Flt-1 | Mild (p-glucose ≥ 6.0 and <11 mmol/L) | 1.11 | 0.98 | 1.25 | 0.096 | 0.582 |
|  | Severe (p-glucose ≥ 11.0 mmol/L) | 1.22 | 1.03 | 1.44 | 0.024 | 0.335 |
| GM-CSF | Mild (p-glucose ≥ 6.0 and <11 mmol/L) | 0.97 | 0.92 | 1.03 | 0.309 | 0.911 |
|  | Severe (p-glucose ≥ 11.0 mmol/L) | 0.99 | 0.92 | 1.07 | 0.869 | 0.980 |
| IFN-γ | Mild (p-glucose ≥ 6.0 and <11 mmol/L) | 1.11 | 0.78 | 1.57 | 0.575 | 0.980 |
|  | Severe (p-glucose ≥ 11.0 mmol/L) | 1.06 | 0.64 | 1.75 | 0.819 | 0.980 |
| IL-10 | Mild (p-glucose ≥ 6.0 and <11 mmol/L) | 1.02 | 0.83 | 1.26 | 0.817 | 0.980 |
|  | Severe (p-glucose ≥ 11.0 mmol/L) | 0.99 | 0.73 | 1.33 | 0.921 | 0.980 |
| IL-12 | Mild (p-glucose ≥ 6.0 and <11 mmol/L) | 0.90 | 0.73 | 1.11 | 0.330 | 0.911 |
|  | Severe (p-glucose ≥ 11.0 mmol/L) | 0.99 | 0.73 | 1.33 | 0.923 | 0.980 |
| IL-12p70 | Mild (p-glucose ≥ 6.0 and <11 mmol/L) | 0.99 | 0.92 | 1.07 | 0.887 | 0.980 |
|  | Severe (p-glucose ≥ 11.0 mmol/L) | 0.98 | 0.88 | 1.09 | 0.738 | 0.980 |
| IL-13 | Mild (p-glucose ≥ 6.0 and <11 mmol/L) | 1.06 | 0.99 | 1.14 | 0.083 | 0.547 |
|  | Severe (p-glucose ≥ 11.0 mmol/L) | 0.98 | 0.89 | 1.08 | 0.730 | 0.980 |
| IL-15 | Mild (p-glucose ≥ 6.0 and <11 mmol/L) | 1.11 | 1.00 | 1.23 | 0.047 | 0.430 |
|  | Severe (p-glucose ≥ 11.0 mmol/L) | 1.34 | 1.16 | 1.55 | 0.000 | 0.073 |
| IL-16 | Mild (p-glucose ≥ 6.0 and <11 mmol/L) | 1.06 | 0.93 | 1.20 | 0.373 | 0.918 |
|  | Severe (p-glucose ≥ 11.0 mmol/L) | 1.27 | 1.05 | 1.52 | 0.012 | 0.316 |
| IL-17A | Mild (p-glucose ≥ 6.0 and <11 mmol/L) | 0.98 | 0.82 | 1.17 | 0.805 | 0.980 |
|  | Severe (p-glucose ≥ 11.0 mmol/L) | 1.22 | 0.95 | 1.57 | 0.119 | 0.634 |
| IL-17A/F | Mild (p-glucose ≥ 6.0 and <11 mmol/L) | 1.03 | 0.95 | 1.13 | 0.444 | 0.957 |
|  | Severe (p-glucose ≥ 11.0 mmol/L) | 1.08 | 0.95 | 1.22 | 0.241 | 0.865 |
| IL-17B | Mild (p-glucose ≥ 6.0 and <11 mmol/L) | 1.00 | 0.91 | 1.10 | 0.986 | 0.995 |
|  | Severe (p-glucose ≥ 11.0 mmol/L) | 0.98 | 0.86 | 1.12 | 0.744 | 0.980 |
| IL-17C | Mild (p-glucose ≥ 6.0 and <11 mmol/L) | 0.94 | 0.86 | 1.03 | 0.162 | 0.742 |
|  | Severe (p-glucose ≥ 11.0 mmol/L) | 1.03 | 0.91 | 1.17 | 0.607 | 0.980 |
| IL-17D | Mild (p-glucose ≥ 6.0 and <11 mmol/L) | 1.02 | 0.95 | 1.10 | 0.615 | 0.980 |
|  | Severe (p-glucose ≥ 11.0 mmol/L) | 1.03 | 0.93 | 1.14 | 0.589 | 0.980 |
| IL-1RA | Mild (p-glucose ≥ 6.0 and <11 mmol/L) | 1.25 | 0.93 | 1.68 | 0.138 | 0.691 |
|  | Severe (p-glucose ≥ 11.0 mmol/L) | 1.83 | 1.20 | 2.78 | 0.005 | 0.316 |
| IL-1α | Mild (p-glucose ≥ 6.0 and <11 mmol/L) | 0.95 | 0.86 | 1.05 | 0.310 | 0.911 |
|  | Severe (p-glucose ≥ 11.0 mmol/L) | 0.87 | 0.75 | 1.01 | 0.070 | 0.509 |
| IL-1β | Mild (p-glucose ≥ 6.0 and <11 mmol/L) | 1.00 | 0.92 | 1.08 | 0.955 | 0.982 |
|  | Severe (p-glucose ≥ 11.0 mmol/L) | 0.96 | 0.86 | 1.07 | 0.439 | 0.957 |
| IL-2 | Mild (p-glucose ≥ 6.0 and <11 mmol/L) | 1.02 | 0.92 | 1.13 | 0.687 | 0.980 |
|  | Severe (p-glucose ≥ 11.0 mmol/L) | 1.08 | 0.93 | 1.25 | 0.329 | 0.911 |
| IL-3 | Mild (p-glucose ≥ 6.0 and <11 mmol/L) | 1.03 | 0.92 | 1.15 | 0.595 | 0.980 |
|  | Severe (p-glucose ≥ 11.0 mmol/L) | 1.02 | 0.87 | 1.20 | 0.776 | 0.980 |
| IL-4 | Mild (p-glucose ≥ 6.0 and <11 mmol/L) | 0.99 | 0.90 | 1.09 | 0.844 | 0.980 |
|  | Severe (p-glucose ≥ 11.0 mmol/L) | 1.01 | 0.88 | 1.15 | 0.901 | 0.980 |
| IL-5 | Mild (p-glucose ≥ 6.0 and <11 mmol/L) | 0.96 | 0.80 | 1.15 | 0.680 | 0.980 |
|  | Severe (p-glucose ≥ 11.0 mmol/L) | 0.88 | 0.68 | 1.14 | 0.332 | 0.911 |
| IL-6 | Mild (p-glucose ≥ 6.0 and <11 mmol/L) | 1.06 | 0.76 | 1.49 | 0.714 | 0.980 |
|  | Severe (p-glucose ≥ 11.0 mmol/L) | 1.04 | 0.64 | 1.68 | 0.882 | 0.980 |
| IL-7 | Mild (p-glucose ≥ 6.0 and <11 mmol/L) | 1.06 | 0.91 | 1.23 | 0.438 | 0.957 |
|  | Severe (p-glucose ≥ 11.0 mmol/L) | 0.99 | 0.80 | 1.23 | 0.947 | 0.980 |
| IL-8 | Mild (p-glucose ≥ 6.0 and <11 mmol/L) | 1.01 | 0.94 | 1.09 | 0.797 | 0.980 |
|  | Severe (p-glucose ≥ 11.0 mmol/L) | 1.11 | 1.00 | 1.23 | 0.052 | 0.443 |
| IL-9 | Mild (p-glucose ≥ 6.0 and <11 mmol/L) | 1.03 | 0.90 | 1.17 | 0.696 | 0.980 |
|  | Severe (p-glucose ≥ 11.0 mmol/L) | 1.02 | 0.85 | 1.23 | 0.802 | 0.980 |
| IP-10 | Mild (p-glucose ≥ 6.0 and <11 mmol/L) | 1.13 | 0.87 | 1.48 | 0.366 | 0.912 |
|  | Severe (p-glucose ≥ 11.0 mmol/L) | 1.08 | 0.74 | 1.58 | 0.696 | 0.980 |
| MCP-1 | Mild (p-glucose ≥ 6.0 and <11 mmol/L) | 0.94 | 0.79 | 1.12 | 0.487 | 0.967 |
|  | Severe (p-glucose ≥ 11.0 mmol/L) | 1.08 | 0.84 | 1.38 | 0.573 | 0.980 |
| MCP-4 | Mild (p-glucose ≥ 6.0 and <11 mmol/L) | 0.92 | 0.78 | 1.07 | 0.270 | 0.897 |
|  | Severe (p-glucose ≥ 11.0 mmol/L) | 0.93 | 0.74 | 1.16 | 0.512 | 0.969 |
| MDC | Mild (p-glucose ≥ 6.0 and <11 mmol/L) | 0.87 | 0.75 | 1.00 | 0.054 | 0.450 |
|  | Severe (p-glucose ≥ 11.0 mmol/L) | 0.79 | 0.64 | 0.97 | 0.023 | 0.335 |
| MIP-1α | Mild (p-glucose ≥ 6.0 and <11 mmol/L) | 1.02 | 0.90 | 1.16 | 0.760 | 0.980 |
|  | Severe (p-glucose ≥ 11.0 mmol/L) | 1.02 | 0.85 | 1.22 | 0.803 | 0.980 |
| MIP-1β | Mild (p-glucose ≥ 6.0 and <11 mmol/L) | 1.13 | 0.92 | 1.40 | 0.250 | 0.868 |
|  | Severe (p-glucose ≥ 11.0 mmol/L) | 1.09 | 0.81 | 1.48 | 0.567 | 0.980 |
| PlGF | Mild (p-glucose ≥ 6.0 and <11 mmol/L) | 1.02 | 0.94 | 1.10 | 0.671 | 0.980 |
|  | Severe (p-glucose ≥ 11.0 mmol/L) | 0.97 | 0.86 | 1.08 | 0.554 | 0.980 |
| SAA | Mild (p-glucose ≥ 6.0 and <11 mmol/L) | 1.25 | 1.04 | 1.51 | 0.015 | 0.335 |
|  | Severe (p-glucose ≥ 11.0 mmol/L) | 1.27 | 0.98 | 1.65 | 0.072 | 0.510 |
| TARC | Mild (p-glucose ≥ 6.0 and <11 mmol/L) | 0.97 | 0.79 | 1.20 | 0.799 | 0.980 |
|  | Severe (p-glucose ≥ 11.0 mmol/L) | 0.81 | 0.60 | 1.10 | 0.174 | 0.751 |
| TLSP | Mild (p-glucose ≥ 6.0 and <11 mmol/L) | 0.96 | 0.83 | 1.12 | 0.605 | 0.980 |
|  | Severe (p-glucose ≥ 11.0 mmol/L) | 0.97 | 0.79 | 1.20 | 0.811 | 0.980 |
| TNF-α | Mild (p-glucose ≥ 6.0 and <11 mmol/L) | 0.96 | 0.83 | 1.10 | 0.549 | 0.980 |
|  | Severe (p-glucose ≥ 11.0 mmol/L) | 1.00 | 0.82 | 1.22 | 0.984 | 0.995 |
| TNF-β | Mild (p-glucose ≥ 6.0 and <11 mmol/L) | 0.99 | 0.95 | 1.04 | 0.690 | 0.980 |
|  | Severe (p-glucose ≥ 11.0 mmol/L) | 1.00 | 0.93 | 1.06 | 0.897 | 0.980 |
| Tie-2 | Mild (p-glucose ≥ 6.0 and <11 mmol/L) | 1.03 | 0.96 | 1.09 | 0.434 | 0.957 |
|  | Severe (p-glucose ≥ 11.0 mmol/L) | 1.11 | 1.02 | 1.22 | 0.021 | 0.335 |
| VEGF-A | Mild (p-glucose ≥ 6.0 and <11 mmol/L) | 0.99 | 0.80 | 1.24 | 0.950 | 0.980 |
|  | Severe (p-glucose ≥ 11.0 mmol/L) | 0.96 | 0.70 | 1.31 | 0.810 | 0.980 |
| VEGF-C | Mild (p-glucose ≥ 6.0 and <11 mmol/L) | 1.01 | 0.92 | 1.11 | 0.830 | 0.980 |
|  | Severe (p-glucose ≥ 11.0 mmol/L) | 1.09 | 0.95 | 1.25 | 0.204 | 0.808 |
| VEGF-D | Mild (p-glucose ≥ 6.0 and <11 mmol/L) | 0.95 | 0.85 | 1.06 | 0.364 | 0.912 |
|  | Severe (p-glucose ≥ 11.0 mmol/L) | 1.06 | 0.90 | 1.24 | 0.496 | 0.967 |
| bFGF | Mild (p-glucose ≥ 6.0 and <11 mmol/L) | 0.93 | 0.74 | 1.17 | 0.530 | 0.980 |
|  | Severe (p-glucose ≥ 11.0 mmol/L) | 0.96 | 0.69 | 1.32 | 0.791 | 0.980 |
| sICAM-1 | Mild (p-glucose ≥ 6.0 and <11 mmol/L) | 0.98 | 0.90 | 1.07 | 0.724 | 0.980 |
|  | Severe (p-glucose ≥ 11.0 mmol/L) | 1.02 | 0.90 | 1.15 | 0.805 | 0.980 |
| sVCAM-1 | Mild (p-glucose ≥ 6.0 and <11 mmol/L) | 1.03 | 0.94 | 1.11 | 0.560 | 0.980 |
|  | Severe (p-glucose ≥ 11.0 mmol/L) | 1.13 | 1.00 | 1.27 | 0.050 | 0.443 |
| Abbreviations: CI, confidence interval, FDR, false discovery rate | | | | | | |

| Supplementary Table 9: Summaries of adjusted models with acute hyperglycaemia as the predictor  Euglycaemia (admission p-glucose < 6.0 mmol/L) was the reference for each comparison | | | | | | |
| --- | --- | --- | --- | --- | --- | --- |
| Analyte | Predictor | Estimate | 95% CI lower | 95% CI higher | P-value | FDR-adjusted p-value |
| CRP | Mild (p-glucose ≥ 6.0 and <11 mmol/L) | 1.17 | 0.99 | 1.37 | 0.058 | 0.467 |
|  | Severe (p-glucose ≥ 11.0 mmol/L) | 1.18 | 0.91 | 1.54 | 0.210 | 0.811 |
| Eotaxin | Mild (p-glucose ≥ 6.0 and <11 mmol/L) | 0.99 | 0.84 | 1.17 | 0.895 | 0.980 |
|  | Severe (p-glucose ≥ 11.0 mmol/L) | 1.08 | 0.82 | 1.42 | 0.582 | 0.980 |
| Eotaxin-3 | Mild (p-glucose ≥ 6.0 and <11 mmol/L) | 1.05 | 0.85 | 1.30 | 0.648 | 0.980 |
|  | Severe (p-glucose ≥ 11.0 mmol/L) | 0.85 | 0.60 | 1.20 | 0.355 | 0.911 |
| Flt-1 | Mild (p-glucose ≥ 6.0 and <11 mmol/L) | 1.11 | 0.97 | 1.26 | 0.122 | 0.636 |
|  | Severe (p-glucose ≥ 11.0 mmol/L) | 1.10 | 0.89 | 1.36 | 0.375 | 0.918 |
| GM-CSF | Mild (p-glucose ≥ 6.0 and <11 mmol/L) | 0.98 | 0.92 | 1.04 | 0.442 | 0.957 |
|  | Severe (p-glucose ≥ 11.0 mmol/L) | 1.02 | 0.93 | 1.12 | 0.682 | 0.980 |
| IFN-γ | Mild (p-glucose ≥ 6.0 and <11 mmol/L) | 1.34 | 0.93 | 1.94 | 0.118 | 0.634 |
|  | Severe (p-glucose ≥ 11.0 mmol/L) | 1.14 | 0.62 | 2.08 | 0.677 | 0.980 |
| IL-10 | Mild (p-glucose ≥ 6.0 and <11 mmol/L) | 1.10 | 0.88 | 1.39 | 0.398 | 0.936 |
|  | Severe (p-glucose ≥ 11.0 mmol/L) | 1.09 | 0.75 | 1.58 | 0.659 | 0.980 |
| IL-12 | Mild (p-glucose ≥ 6.0 and <11 mmol/L) | 0.94 | 0.76 | 1.18 | 0.611 | 0.980 |
|  | Severe (p-glucose ≥ 11.0 mmol/L) | 0.82 | 0.57 | 1.19 | 0.300 | 0.911 |
| IL-12p70 | Mild (p-glucose ≥ 6.0 and <11 mmol/L) | 1.00 | 0.92 | 1.08 | 0.924 | 0.980 |
|  | Severe (p-glucose ≥ 11.0 mmol/L) | 0.98 | 0.87 | 1.12 | 0.806 | 0.980 |
| IL-13 | Mild (p-glucose ≥ 6.0 and <11 mmol/L) | 1.07 | 0.99 | 1.15 | 0.086 | 0.549 |
|  | Severe (p-glucose ≥ 11.0 mmol/L) | 0.99 | 0.87 | 1.12 | 0.900 | 0.980 |
| IL-15 | Mild (p-glucose ≥ 6.0 and <11 mmol/L) | 1.13 | 1.01 | 1.27 | 0.036 | 0.383 |
|  | Severe (p-glucose ≥ 11.0 mmol/L) | 1.33 | 1.10 | 1.61 | 0.003 | 0.308 |
| IL-16 | Mild (p-glucose ≥ 6.0 and <11 mmol/L) | 1.04 | 0.90 | 1.20 | 0.569 | 0.980 |
|  | Severe (p-glucose ≥ 11.0 mmol/L) | 1.16 | 0.92 | 1.47 | 0.208 | 0.811 |
| IL-17A | Mild (p-glucose ≥ 6.0 and <11 mmol/L) | 0.94 | 0.78 | 1.13 | 0.480 | 0.960 |
|  | Severe (p-glucose ≥ 11.0 mmol/L) | 1.06 | 0.79 | 1.43 | 0.695 | 0.980 |
| IL-17A/F | Mild (p-glucose ≥ 6.0 and <11 mmol/L) | 1.02 | 0.92 | 1.13 | 0.723 | 0.980 |
|  | Severe (p-glucose ≥ 11.0 mmol/L) | 1.05 | 0.88 | 1.24 | 0.590 | 0.980 |
| IL-17B | Mild (p-glucose ≥ 6.0 and <11 mmol/L) | 1.01 | 0.92 | 1.11 | 0.877 | 0.980 |
|  | Severe (p-glucose ≥ 11.0 mmol/L) | 0.94 | 0.81 | 1.10 | 0.457 | 0.957 |
| IL-17C | Mild (p-glucose ≥ 6.0 and <11 mmol/L) | 0.90 | 0.81 | 0.99 | 0.032 | 0.376 |
|  | Severe (p-glucose ≥ 11.0 mmol/L) | 0.92 | 0.78 | 1.08 | 0.313 | 0.911 |
| IL-17D | Mild (p-glucose ≥ 6.0 and <11 mmol/L) | 0.99 | 0.91 | 1.08 | 0.812 | 0.980 |
|  | Severe (p-glucose ≥ 11.0 mmol/L) | 0.95 | 0.83 | 1.09 | 0.486 | 0.967 |
| IL-1RA | Mild (p-glucose ≥ 6.0 and <11 mmol/L) | 1.27 | 0.94 | 1.73 | 0.118 | 0.634 |
|  | Severe (p-glucose ≥ 11.0 mmol/L) | 1.73 | 1.05 | 2.84 | 0.031 | 0.376 |
| IL-1α | Mild (p-glucose ≥ 6.0 and <11 mmol/L) | 0.95 | 0.85 | 1.07 | 0.412 | 0.946 |
|  | Severe (p-glucose ≥ 11.0 mmol/L) | 0.83 | 0.69 | 1.01 | 0.058 | 0.467 |
| IL-1β | Mild (p-glucose ≥ 6.0 and <11 mmol/L) | 0.99 | 0.91 | 1.08 | 0.857 | 0.980 |
|  | Severe (p-glucose ≥ 11.0 mmol/L) | 0.99 | 0.86 | 1.15 | 0.942 | 0.980 |
| IL-2 | Mild (p-glucose ≥ 6.0 and <11 mmol/L) | 1.09 | 0.97 | 1.21 | 0.152 | 0.724 |
|  | Severe (p-glucose ≥ 11.0 mmol/L) | 1.12 | 0.93 | 1.35 | 0.220 | 0.819 |
| IL-3 | Mild (p-glucose ≥ 6.0 and <11 mmol/L) | 1.00 | 0.88 | 1.14 | 0.998 | 0.999 |
|  | Severe (p-glucose ≥ 11.0 mmol/L) | 1.07 | 0.87 | 1.33 | 0.504 | 0.967 |
| IL-4 | Mild (p-glucose ≥ 6.0 and <11 mmol/L) | 0.99 | 0.90 | 1.09 | 0.831 | 0.980 |
|  | Severe (p-glucose ≥ 11.0 mmol/L) | 0.98 | 0.84 | 1.14 | 0.786 | 0.980 |
| IL-5 | Mild (p-glucose ≥ 6.0 and <11 mmol/L) | 1.03 | 0.84 | 1.26 | 0.794 | 0.980 |
|  | Severe (p-glucose ≥ 11.0 mmol/L) | 0.85 | 0.62 | 1.19 | 0.347 | 0.911 |
| IL-6 | Mild (p-glucose ≥ 6.0 and <11 mmol/L) | 1.11 | 0.79 | 1.54 | 0.554 | 0.980 |
|  | Severe (p-glucose ≥ 11.0 mmol/L) | 0.91 | 0.53 | 1.58 | 0.748 | 0.980 |
| IL-7 | Mild (p-glucose ≥ 6.0 and <11 mmol/L) | 1.05 | 0.90 | 1.23 | 0.545 | 0.980 |
|  | Severe (p-glucose ≥ 11.0 mmol/L) | 0.97 | 0.74 | 1.25 | 0.788 | 0.980 |
| IL-8 | Mild (p-glucose ≥ 6.0 and <11 mmol/L) | 1.00 | 0.93 | 1.09 | 0.908 | 0.980 |
|  | Severe (p-glucose ≥ 11.0 mmol/L) | 1.14 | 1.00 | 1.29 | 0.050 | 0.443 |
| IL-9 | Mild (p-glucose ≥ 6.0 and <11 mmol/L) | 1.00 | 0.85 | 1.16 | 0.949 | 0.980 |
|  | Severe (p-glucose ≥ 11.0 mmol/L) | 0.95 | 0.74 | 1.22 | 0.689 | 0.980 |
| IP-10 | Mild (p-glucose ≥ 6.0 and <11 mmol/L) | 1.27 | 0.95 | 1.70 | 0.106 | 0.605 |
|  | Severe (p-glucose ≥ 11.0 mmol/L) | 1.15 | 0.72 | 1.85 | 0.555 | 0.980 |
| MCP-1 | Mild (p-glucose ≥ 6.0 and <11 mmol/L) | 0.93 | 0.77 | 1.13 | 0.465 | 0.957 |
|  | Severe (p-glucose ≥ 11.0 mmol/L) | 1.04 | 0.76 | 1.42 | 0.806 | 0.980 |
| MCP-4 | Mild (p-glucose ≥ 6.0 and <11 mmol/L) | 0.88 | 0.73 | 1.04 | 0.140 | 0.691 |
|  | Severe (p-glucose ≥ 11.0 mmol/L) | 0.83 | 0.62 | 1.11 | 0.203 | 0.808 |
| MDC | Mild (p-glucose ≥ 6.0 and <11 mmol/L) | 0.83 | 0.71 | 0.97 | 0.021 | 0.335 |
|  | Severe (p-glucose ≥ 11.0 mmol/L) | 0.70 | 0.54 | 0.92 | 0.009 | 0.316 |
| MIP-1α | Mild (p-glucose ≥ 6.0 and <11 mmol/L) | 1.00 | 0.88 | 1.14 | 0.960 | 0.984 |
|  | Severe (p-glucose ≥ 11.0 mmol/L) | 1.04 | 0.85 | 1.29 | 0.686 | 0.980 |
| MIP-1β | Mild (p-glucose ≥ 6.0 and <11 mmol/L) | 1.11 | 0.90 | 1.37 | 0.328 | 0.911 |
|  | Severe (p-glucose ≥ 11.0 mmol/L) | 1.03 | 0.73 | 1.45 | 0.868 | 0.980 |
| PlGF | Mild (p-glucose ≥ 6.0 and <11 mmol/L) | 1.03 | 0.95 | 1.13 | 0.443 | 0.957 |
|  | Severe (p-glucose ≥ 11.0 mmol/L) | 1.02 | 0.89 | 1.17 | 0.808 | 0.980 |
| SAA | Mild (p-glucose ≥ 6.0 and <11 mmol/L) | 1.17 | 0.96 | 1.42 | 0.127 | 0.656 |
|  | Severe (p-glucose ≥ 11.0 mmol/L) | 1.35 | 0.98 | 1.86 | 0.070 | 0.509 |
| TARC | Mild (p-glucose ≥ 6.0 and <11 mmol/L) | 0.97 | 0.77 | 1.23 | 0.815 | 0.980 |
|  | Severe (p-glucose ≥ 11.0 mmol/L) | 0.66 | 0.44 | 0.97 | 0.034 | 0.376 |
| TLSP | Mild (p-glucose ≥ 6.0 and <11 mmol/L) | 0.93 | 0.79 | 1.09 | 0.354 | 0.911 |
|  | Severe (p-glucose ≥ 11.0 mmol/L) | 0.95 | 0.73 | 1.24 | 0.709 | 0.980 |
| TNF-α | Mild (p-glucose ≥ 6.0 and <11 mmol/L) | 0.93 | 0.81 | 1.07 | 0.307 | 0.911 |
|  | Severe (p-glucose ≥ 11.0 mmol/L) | 0.94 | 0.75 | 1.18 | 0.609 | 0.980 |
| TNF-β | Mild (p-glucose ≥ 6.0 and <11 mmol/L) | 1.00 | 0.95 | 1.05 | 0.917 | 0.980 |
|  | Severe (p-glucose ≥ 11.0 mmol/L) | 1.00 | 0.92 | 1.09 | 0.932 | 0.980 |
| Tie-2 | Mild (p-glucose ≥ 6.0 and <11 mmol/L) | 1.03 | 0.95 | 1.11 | 0.464 | 0.957 |
|  | Severe (p-glucose ≥ 11.0 mmol/L) | 1.07 | 0.94 | 1.20 | 0.304 | 0.911 |
| VEGF-A | Mild (p-glucose ≥ 6.0 and <11 mmol/L) | 0.93 | 0.73 | 1.17 | 0.517 | 0.969 |
|  | Severe (p-glucose ≥ 11.0 mmol/L) | 0.89 | 0.61 | 1.30 | 0.540 | 0.980 |
| VEGF-C | Mild (p-glucose ≥ 6.0 and <11 mmol/L) | 0.99 | 0.88 | 1.10 | 0.802 | 0.980 |
|  | Severe (p-glucose ≥ 11.0 mmol/L) | 1.05 | 0.88 | 1.25 | 0.610 | 0.980 |
| VEGF-D | Mild (p-glucose ≥ 6.0 and <11 mmol/L) | 0.95 | 0.84 | 1.08 | 0.444 | 0.957 |
|  | Severe (p-glucose ≥ 11.0 mmol/L) | 1.08 | 0.88 | 1.32 | 0.466 | 0.957 |
| bFGF | Mild (p-glucose ≥ 6.0 and <11 mmol/L) | 0.90 | 0.70 | 1.15 | 0.410 | 0.942 |
|  | Severe (p-glucose ≥ 11.0 mmol/L) | 0.76 | 0.51 | 1.14 | 0.188 | 0.791 |
| sICAM-1 | Mild (p-glucose ≥ 6.0 and <11 mmol/L) | 0.98 | 0.89 | 1.08 | 0.703 | 0.980 |
|  | Severe (p-glucose ≥ 11.0 mmol/L) | 0.99 | 0.85 | 1.16 | 0.919 | 0.980 |
| sVCAM-1 | Mild (p-glucose ≥ 6.0 and <11 mmol/L) | 1.01 | 0.92 | 1.11 | 0.821 | 0.980 |
|  | Severe (p-glucose ≥ 11.0 mmol/L) | 0.97 | 0.83 | 1.13 | 0.688 | 0.980 |
| Abbreviations: CI, confidence interval, FDR, false discovery rate | | | | | | |

| Supplementary Table 10: Summaries of base models with glycaemic gap quartiles as the predictor  First quartile was the reference for each comparison | | | | | | |
| --- | --- | --- | --- | --- | --- | --- |
| Analyte | Predictor | Estimate | 95% CI lower | 95% CI higher | P-value | FDR-adjusted p-value |
| CRP | Second quartile | 1.20 | 1.03 | 1.39 | 0.021 | 0.335 |
|  | Third quartile | 1.20 | 1.03 | 1.39 | 0.022 | 0.335 |
|  | Fourth quartile | 1.15 | 0.99 | 1.34 | 0.072 | 0.510 |
| Eotaxin | Second quartile | 0.89 | 0.77 | 1.05 | 0.161 | 0.742 |
|  | Third quartile | 0.80 | 0.69 | 0.94 | 0.006 | 0.316 |
|  | Fourth quartile | 0.92 | 0.79 | 1.07 | 0.285 | 0.911 |
| Eotaxin-3 | Second quartile | 1.05 | 0.85 | 1.29 | 0.659 | 0.980 |
|  | Third quartile | 0.90 | 0.73 | 1.11 | 0.337 | 0.911 |
|  | Fourth quartile | 1.00 | 0.81 | 1.23 | 0.990 | 0.995 |
| Flt-1 | Second quartile | 1.01 | 0.89 | 1.15 | 0.860 | 0.980 |
|  | Third quartile | 0.98 | 0.87 | 1.12 | 0.798 | 0.980 |
|  | Fourth quartile | 1.15 | 1.02 | 1.31 | 0.026 | 0.336 |
| GM-CSF | Second quartile | 0.98 | 0.92 | 1.03 | 0.398 | 0.936 |
|  | Third quartile | 0.99 | 0.93 | 1.04 | 0.640 | 0.980 |
|  | Fourth quartile | 1.01 | 0.95 | 1.06 | 0.843 | 0.980 |
| IFN-γ | Second quartile | 0.79 | 0.55 | 1.15 | 0.215 | 0.813 |
|  | Third quartile | 0.92 | 0.63 | 1.33 | 0.644 | 0.980 |
|  | Fourth quartile | 0.87 | 0.60 | 1.26 | 0.449 | 0.957 |
| IL-10 | Second quartile | 0.92 | 0.74 | 1.14 | 0.437 | 0.957 |
|  | Third quartile | 1.09 | 0.87 | 1.35 | 0.459 | 0.957 |
|  | Fourth quartile | 1.11 | 0.89 | 1.38 | 0.372 | 0.917 |
| IL-12 | Second quartile | 0.92 | 0.73 | 1.15 | 0.451 | 0.957 |
|  | Third quartile | 0.82 | 0.65 | 1.02 | 0.074 | 0.515 |
|  | Fourth quartile | 0.86 | 0.69 | 1.08 | 0.198 | 0.806 |
| IL-12p70 | Second quartile | 0.99 | 0.92 | 1.07 | 0.898 | 0.980 |
|  | Third quartile | 1.06 | 0.98 | 1.14 | 0.168 | 0.744 |
|  | Fourth quartile | 1.05 | 0.97 | 1.14 | 0.198 | 0.806 |
| IL-13 | Second quartile | 1.09 | 1.01 | 1.17 | 0.022 | 0.335 |
|  | Third quartile | 1.10 | 1.02 | 1.18 | 0.012 | 0.316 |
|  | Fourth quartile | 1.04 | 0.97 | 1.12 | 0.243 | 0.867 |
| IL-15 | Second quartile | 0.96 | 0.86 | 1.07 | 0.466 | 0.957 |
|  | Third quartile | 0.99 | 0.88 | 1.10 | 0.790 | 0.980 |
|  | Fourth quartile | 1.14 | 1.02 | 1.27 | 0.020 | 0.335 |
| IL-16 | Second quartile | 0.98 | 0.86 | 1.12 | 0.762 | 0.980 |
|  | Third quartile | 0.86 | 0.75 | 0.98 | 0.024 | 0.335 |
|  | Fourth quartile | 1.07 | 0.94 | 1.23 | 0.317 | 0.911 |
| IL-17A | Second quartile | 0.91 | 0.75 | 1.10 | 0.327 | 0.911 |
|  | Third quartile | 1.00 | 0.83 | 1.21 | 0.980 | 0.995 |
|  | Fourth quartile | 1.03 | 0.85 | 1.24 | 0.795 | 0.980 |
| IL-17A/F | Second quartile | 1.01 | 0.92 | 1.10 | 0.899 | 0.980 |
|  | Third quartile | 1.04 | 0.95 | 1.14 | 0.420 | 0.949 |
|  | Fourth quartile | 1.10 | 1.01 | 1.21 | 0.033 | 0.376 |
| IL-17B | Second quartile | 0.93 | 0.84 | 1.03 | 0.143 | 0.692 |
|  | Third quartile | 0.98 | 0.89 | 1.08 | 0.667 | 0.980 |
|  | Fourth quartile | 0.99 | 0.90 | 1.09 | 0.865 | 0.980 |
| IL-17C | Second quartile | 0.91 | 0.83 | 1.00 | 0.043 | 0.407 |
|  | Third quartile | 0.97 | 0.88 | 1.06 | 0.448 | 0.957 |
|  | Fourth quartile | 1.11 | 1.01 | 1.22 | 0.025 | 0.335 |
| IL-17D | Second quartile | 0.90 | 0.83 | 0.98 | 0.010 | 0.316 |
|  | Third quartile | 0.97 | 0.90 | 1.05 | 0.496 | 0.967 |
|  | Fourth quartile | 1.00 | 0.93 | 1.09 | 0.927 | 0.980 |
| IL-1RA | Second quartile | 0.87 | 0.64 | 1.19 | 0.382 | 0.926 |
|  | Third quartile | 0.85 | 0.62 | 1.16 | 0.298 | 0.911 |
|  | Fourth quartile | 1.27 | 0.93 | 1.73 | 0.138 | 0.691 |
| IL-1α | Second quartile | 1.03 | 0.92 | 1.14 | 0.651 | 0.980 |
|  | Third quartile | 1.10 | 0.99 | 1.23 | 0.079 | 0.535 |
|  | Fourth quartile | 0.96 | 0.86 | 1.07 | 0.488 | 0.967 |
| IL-1β | Second quartile | 0.99 | 0.91 | 1.07 | 0.742 | 0.980 |
|  | Third quartile | 1.02 | 0.94 | 1.11 | 0.573 | 0.980 |
|  | Fourth quartile | 1.02 | 0.94 | 1.10 | 0.673 | 0.980 |
| IL-2 | Second quartile | 0.99 | 0.89 | 1.10 | 0.841 | 0.980 |
|  | Third quartile | 1.13 | 1.01 | 1.25 | 0.033 | 0.376 |
|  | Fourth quartile | 1.08 | 0.97 | 1.20 | 0.161 | 0.742 |
| IL-3 | Second quartile | 0.96 | 0.86 | 1.08 | 0.525 | 0.978 |
|  | Third quartile | 1.00 | 0.89 | 1.12 | 0.941 | 0.980 |
|  | Fourth quartile | 1.04 | 0.92 | 1.16 | 0.546 | 0.980 |
| IL-4 | Second quartile | 1.03 | 0.93 | 1.13 | 0.603 | 0.980 |
|  | Third quartile | 1.13 | 1.03 | 1.25 | 0.013 | 0.316 |
|  | Fourth quartile | 1.06 | 0.96 | 1.17 | 0.251 | 0.868 |
| IL-5 | Second quartile | 1.03 | 0.85 | 1.25 | 0.736 | 0.980 |
|  | Third quartile | 0.98 | 0.81 | 1.19 | 0.840 | 0.980 |
|  | Fourth quartile | 0.89 | 0.73 | 1.07 | 0.211 | 0.811 |
| IL-6 | Second quartile | 1.03 | 0.72 | 1.47 | 0.877 | 0.980 |
|  | Third quartile | 1.14 | 0.80 | 1.62 | 0.476 | 0.960 |
|  | Fourth quartile | 1.28 | 0.90 | 1.83 | 0.173 | 0.750 |
| IL-7 | Second quartile | 1.06 | 0.90 | 1.24 | 0.479 | 0.960 |
|  | Third quartile | 1.01 | 0.87 | 1.18 | 0.877 | 0.980 |
|  | Fourth quartile | 0.95 | 0.81 | 1.11 | 0.478 | 0.960 |
| IL-8 | Second quartile | 0.99 | 0.91 | 1.07 | 0.733 | 0.980 |
|  | Third quartile | 0.94 | 0.87 | 1.02 | 0.119 | 0.634 |
|  | Fourth quartile | 1.03 | 0.96 | 1.12 | 0.401 | 0.936 |
| IL-9 | Second quartile | 0.92 | 0.81 | 1.06 | 0.252 | 0.868 |
|  | Third quartile | 0.96 | 0.83 | 1.09 | 0.508 | 0.967 |
|  | Fourth quartile | 0.97 | 0.84 | 1.11 | 0.619 | 0.980 |
| IP-10 | Second quartile | 0.97 | 0.73 | 1.29 | 0.838 | 0.980 |
|  | Third quartile | 0.80 | 0.60 | 1.06 | 0.120 | 0.634 |
|  | Fourth quartile | 1.02 | 0.77 | 1.35 | 0.885 | 0.980 |
| MCP-1 | Second quartile | 0.99 | 0.82 | 1.19 | 0.893 | 0.980 |
|  | Third quartile | 0.84 | 0.70 | 1.02 | 0.072 | 0.510 |
|  | Fourth quartile | 1.01 | 0.84 | 1.22 | 0.880 | 0.980 |
| MCP-4 | Second quartile | 0.92 | 0.78 | 1.08 | 0.312 | 0.911 |
|  | Third quartile | 0.78 | 0.66 | 0.92 | 0.003 | 0.308 |
|  | Fourth quartile | 0.83 | 0.71 | 0.98 | 0.028 | 0.343 |
| MDC | Second quartile | 0.94 | 0.81 | 1.10 | 0.470 | 0.957 |
|  | Third quartile | 0.80 | 0.69 | 0.94 | 0.005 | 0.316 |
|  | Fourth quartile | 0.82 | 0.71 | 0.96 | 0.013 | 0.316 |
| MIP-1α | Second quartile | 0.94 | 0.83 | 1.08 | 0.386 | 0.927 |
|  | Third quartile | 0.88 | 0.77 | 1.00 | 0.051 | 0.443 |
|  | Fourth quartile | 1.02 | 0.90 | 1.17 | 0.720 | 0.980 |
| MIP-1β | Second quartile | 0.98 | 0.79 | 1.23 | 0.888 | 0.980 |
|  | Third quartile | 0.93 | 0.74 | 1.16 | 0.507 | 0.967 |
|  | Fourth quartile | 1.10 | 0.88 | 1.37 | 0.417 | 0.949 |
| PlGF | Second quartile | 1.01 | 0.93 | 1.10 | 0.768 | 0.980 |
|  | Third quartile | 1.01 | 0.93 | 1.10 | 0.733 | 0.980 |
|  | Fourth quartile | 1.05 | 0.97 | 1.15 | 0.214 | 0.811 |
| SAA | Second quartile | 1.22 | 1.01 | 1.48 | 0.040 | 0.404 |
|  | Third quartile | 1.28 | 1.05 | 1.55 | 0.013 | 0.316 |
|  | Fourth quartile | 1.23 | 1.02 | 1.49 | 0.035 | 0.376 |
| TARC | Second quartile | 0.90 | 0.72 | 1.12 | 0.338 | 0.911 |
|  | Third quartile | 0.86 | 0.69 | 1.07 | 0.168 | 0.744 |
|  | Fourth quartile | 0.81 | 0.65 | 1.02 | 0.068 | 0.506 |
| TLSP | Second quartile | 0.92 | 0.79 | 1.07 | 0.284 | 0.911 |
|  | Third quartile | 0.87 | 0.74 | 1.02 | 0.077 | 0.524 |
|  | Fourth quartile | 1.02 | 0.88 | 1.19 | 0.779 | 0.980 |
| TNF-α | Second quartile | 0.93 | 0.80 | 1.07 | 0.299 | 0.911 |
|  | Third quartile | 0.93 | 0.80 | 1.08 | 0.344 | 0.911 |
|  | Fourth quartile | 0.97 | 0.84 | 1.13 | 0.725 | 0.980 |
| TNF-β | Second quartile | 0.98 | 0.93 | 1.03 | 0.357 | 0.911 |
|  | Third quartile | 0.99 | 0.94 | 1.04 | 0.659 | 0.980 |
|  | Fourth quartile | 0.98 | 0.93 | 1.03 | 0.469 | 0.957 |
| Tie-2 | Second quartile | 1.03 | 0.97 | 1.11 | 0.330 | 0.911 |
|  | Third quartile | 0.98 | 0.92 | 1.05 | 0.639 | 0.980 |
|  | Fourth quartile | 1.05 | 0.98 | 1.12 | 0.164 | 0.742 |
| VEGF-A | Second quartile | 0.97 | 0.77 | 1.22 | 0.807 | 0.980 |
|  | Third quartile | 1.02 | 0.81 | 1.29 | 0.867 | 0.980 |
|  | Fourth quartile | 0.90 | 0.71 | 1.13 | 0.370 | 0.915 |
| VEGF-C | Second quartile | 0.95 | 0.86 | 1.05 | 0.292 | 0.911 |
|  | Third quartile | 0.98 | 0.88 | 1.08 | 0.688 | 0.980 |
|  | Fourth quartile | 0.97 | 0.88 | 1.07 | 0.569 | 0.980 |
| VEGF-D | Second quartile | 0.98 | 0.87 | 1.11 | 0.768 | 0.980 |
|  | Third quartile | 1.00 | 0.88 | 1.13 | 0.961 | 0.984 |
|  | Fourth quartile | 0.99 | 0.88 | 1.12 | 0.928 | 0.980 |
| bFGF | Second quartile | 0.92 | 0.72 | 1.16 | 0.473 | 0.960 |
|  | Third quartile | 0.87 | 0.68 | 1.10 | 0.247 | 0.868 |
|  | Fourth quartile | 0.81 | 0.64 | 1.02 | 0.076 | 0.524 |
| sICAM-1 | Second quartile | 1.06 | 0.97 | 1.16 | 0.218 | 0.818 |
|  | Third quartile | 0.97 | 0.89 | 1.07 | 0.553 | 0.980 |
|  | Fourth quartile | 1.10 | 1.01 | 1.21 | 0.034 | 0.376 |
| sVCAM-1 | Second quartile | 1.00 | 0.92 | 1.09 | 0.989 | 0.995 |
|  | Third quartile | 0.97 | 0.89 | 1.06 | 0.495 | 0.967 |
|  | Fourth quartile | 1.12 | 1.03 | 1.23 | 0.008 | 0.316 |
| Abbreviations: CI, confidence interval, FDR, false discovery rate | | | | | | |

| Supplementary Table 11: Summaries of adjusted models with glycaemic gap quartiles as the predictor  First quartile was the reference for each comparison | | | | | | |
| --- | --- | --- | --- | --- | --- | --- |
| Analyte | Predictor | Estimate | 95% CI lower | 95% CI higher | P-value | FDR-adjusted p-value |
| CRP | Second quartile | 1.21 | 1.03 | 1.44 | 0.025 | 0.335 |
|  | Third quartile | 1.23 | 1.03 | 1.45 | 0.020 | 0.335 |
|  | Fourth quartile | 1.13 | 0.96 | 1.34 | 0.141 | 0.691 |
| Eotaxin | Second quartile | 0.92 | 0.77 | 1.10 | 0.346 | 0.911 |
|  | Third quartile | 0.84 | 0.70 | 1.00 | 0.054 | 0.450 |
|  | Fourth quartile | 0.92 | 0.77 | 1.10 | 0.354 | 0.911 |
| Eotaxin-3 | Second quartile | 1.20 | 0.95 | 1.50 | 0.121 | 0.636 |
|  | Third quartile | 0.97 | 0.78 | 1.23 | 0.827 | 0.980 |
|  | Fourth quartile | 0.98 | 0.79 | 1.23 | 0.893 | 0.980 |
| Flt-1 | Second quartile | 1.02 | 0.89 | 1.17 | 0.781 | 0.980 |
|  | Third quartile | 0.97 | 0.84 | 1.11 | 0.634 | 0.980 |
|  | Fourth quartile | 1.07 | 0.93 | 1.22 | 0.361 | 0.911 |
| GM-CSF | Second quartile | 0.97 | 0.91 | 1.03 | 0.330 | 0.911 |
|  | Third quartile | 0.99 | 0.93 | 1.05 | 0.763 | 0.980 |
|  | Fourth quartile | 1.02 | 0.96 | 1.09 | 0.454 | 0.957 |
| IFN-γ | Second quartile | 0.83 | 0.56 | 1.23 | 0.349 | 0.911 |
|  | Third quartile | 1.03 | 0.69 | 1.52 | 0.896 | 0.980 |
|  | Fourth quartile | 0.82 | 0.55 | 1.21 | 0.309 | 0.911 |
| IL-10 | Second quartile | 0.92 | 0.72 | 1.17 | 0.498 | 0.967 |
|  | Third quartile | 1.16 | 0.91 | 1.47 | 0.237 | 0.865 |
|  | Fourth quartile | 1.10 | 0.87 | 1.40 | 0.432 | 0.957 |
| IL-12 | Second quartile | 0.96 | 0.76 | 1.22 | 0.765 | 0.980 |
|  | Third quartile | 0.82 | 0.64 | 1.03 | 0.091 | 0.567 |
|  | Fourth quartile | 0.82 | 0.65 | 1.03 | 0.089 | 0.556 |
| IL-12p70 | Second quartile | 0.99 | 0.91 | 1.08 | 0.854 | 0.980 |
|  | Third quartile | 1.05 | 0.97 | 1.14 | 0.253 | 0.868 |
|  | Fourth quartile | 1.05 | 0.96 | 1.14 | 0.278 | 0.907 |
| IL-13 | Second quartile | 1.10 | 1.02 | 1.19 | 0.019 | 0.335 |
|  | Third quartile | 1.13 | 1.04 | 1.22 | 0.003 | 0.316 |
|  | Fourth quartile | 1.07 | 0.99 | 1.16 | 0.082 | 0.546 |
| IL-15 | Second quartile | 1.01 | 0.89 | 1.14 | 0.889 | 0.980 |
|  | Third quartile | 1.01 | 0.90 | 1.15 | 0.820 | 0.980 |
|  | Fourth quartile | 1.09 | 0.97 | 1.23 | 0.151 | 0.723 |
| IL-16 | Second quartile | 1.04 | 0.89 | 1.21 | 0.647 | 0.980 |
|  | Third quartile | 0.88 | 0.75 | 1.02 | 0.089 | 0.556 |
|  | Fourth quartile | 1.04 | 0.89 | 1.20 | 0.645 | 0.980 |
| IL-17A | Second quartile | 0.96 | 0.79 | 1.16 | 0.671 | 0.980 |
|  | Third quartile | 1.02 | 0.84 | 1.24 | 0.839 | 0.980 |
|  | Fourth quartile | 0.99 | 0.81 | 1.20 | 0.908 | 0.980 |
| IL-17A/F | Second quartile | 1.00 | 0.89 | 1.11 | 0.945 | 0.980 |
|  | Third quartile | 1.03 | 0.92 | 1.15 | 0.613 | 0.980 |
|  | Fourth quartile | 1.10 | 0.99 | 1.23 | 0.073 | 0.510 |
| IL-17B | Second quartile | 0.92 | 0.83 | 1.01 | 0.086 | 0.549 |
|  | Third quartile | 0.96 | 0.87 | 1.05 | 0.366 | 0.912 |
|  | Fourth quartile | 0.99 | 0.90 | 1.09 | 0.849 | 0.980 |
| IL-17C | Second quartile | 0.90 | 0.81 | 1.00 | 0.056 | 0.455 |
|  | Third quartile | 0.94 | 0.85 | 1.05 | 0.273 | 0.905 |
|  | Fourth quartile | 1.07 | 0.96 | 1.19 | 0.204 | 0.808 |
| IL-17D | Second quartile | 0.88 | 0.80 | 0.96 | 0.004 | 0.316 |
|  | Third quartile | 0.95 | 0.87 | 1.04 | 0.252 | 0.868 |
|  | Fourth quartile | 0.97 | 0.89 | 1.06 | 0.491 | 0.967 |
| IL-1RA | Second quartile | 1.02 | 0.74 | 1.41 | 0.884 | 0.980 |
|  | Third quartile | 0.89 | 0.64 | 1.23 | 0.467 | 0.957 |
|  | Fourth quartile | 1.14 | 0.83 | 1.57 | 0.419 | 0.949 |
| IL-1α | Second quartile | 1.02 | 0.91 | 1.16 | 0.704 | 0.980 |
|  | Third quartile | 1.09 | 0.96 | 1.24 | 0.164 | 0.742 |
|  | Fourth quartile | 0.92 | 0.82 | 1.04 | 0.206 | 0.810 |
| IL-1β | Second quartile | 1.00 | 0.91 | 1.09 | 0.964 | 0.985 |
|  | Third quartile | 1.03 | 0.94 | 1.13 | 0.546 | 0.980 |
|  | Fourth quartile | 1.05 | 0.96 | 1.15 | 0.330 | 0.911 |
| IL-2 | Second quartile | 1.01 | 0.90 | 1.14 | 0.839 | 0.980 |
|  | Third quartile | 1.17 | 1.04 | 1.32 | 0.009 | 0.316 |
|  | Fourth quartile | 1.07 | 0.96 | 1.21 | 0.228 | 0.841 |
| IL-3 | Second quartile | 0.93 | 0.81 | 1.06 | 0.268 | 0.897 |
|  | Third quartile | 0.95 | 0.83 | 1.09 | 0.498 | 0.967 |
|  | Fourth quartile | 1.04 | 0.91 | 1.20 | 0.541 | 0.980 |
| IL-4 | Second quartile | 1.01 | 0.92 | 1.12 | 0.805 | 0.980 |
|  | Third quartile | 1.13 | 1.02 | 1.25 | 0.017 | 0.335 |
|  | Fourth quartile | 1.02 | 0.92 | 1.13 | 0.676 | 0.980 |
| IL-5 | Second quartile | 1.04 | 0.84 | 1.29 | 0.714 | 0.980 |
|  | Third quartile | 1.05 | 0.85 | 1.30 | 0.674 | 0.980 |
|  | Fourth quartile | 0.90 | 0.73 | 1.11 | 0.322 | 0.911 |
| IL-6 | Second quartile | 1.13 | 0.79 | 1.61 | 0.500 | 0.967 |
|  | Third quartile | 1.20 | 0.84 | 1.71 | 0.317 | 0.911 |
|  | Fourth quartile | 1.15 | 0.81 | 1.64 | 0.430 | 0.957 |
| IL-7 | Second quartile | 1.09 | 0.92 | 1.29 | 0.306 | 0.911 |
|  | Third quartile | 1.09 | 0.92 | 1.29 | 0.312 | 0.911 |
|  | Fourth quartile | 0.95 | 0.81 | 1.13 | 0.578 | 0.980 |
| IL-8 | Second quartile | 1.04 | 0.96 | 1.13 | 0.326 | 0.911 |
|  | Third quartile | 0.98 | 0.90 | 1.07 | 0.646 | 0.980 |
|  | Fourth quartile | 1.07 | 0.99 | 1.16 | 0.095 | 0.582 |
| IL-9 | Second quartile | 0.93 | 0.79 | 1.10 | 0.402 | 0.936 |
|  | Third quartile | 0.95 | 0.81 | 1.12 | 0.566 | 0.980 |
|  | Fourth quartile | 0.95 | 0.81 | 1.11 | 0.497 | 0.967 |
| IP-10 | Second quartile | 0.99 | 0.73 | 1.34 | 0.940 | 0.980 |
|  | Third quartile | 0.83 | 0.61 | 1.13 | 0.241 | 0.865 |
|  | Fourth quartile | 0.96 | 0.71 | 1.31 | 0.814 | 0.980 |
| MCP-1 | Second quartile | 1.01 | 0.82 | 1.24 | 0.933 | 0.980 |
|  | Third quartile | 0.85 | 0.70 | 1.05 | 0.130 | 0.667 |
|  | Fourth quartile | 0.97 | 0.79 | 1.19 | 0.773 | 0.980 |
| MCP-4 | Second quartile | 0.97 | 0.80 | 1.16 | 0.724 | 0.980 |
|  | Third quartile | 0.83 | 0.69 | 0.99 | 0.043 | 0.407 |
|  | Fourth quartile | 0.86 | 0.71 | 1.03 | 0.099 | 0.592 |
| MDC | Second quartile | 0.93 | 0.79 | 1.10 | 0.420 | 0.949 |
|  | Third quartile | 0.76 | 0.64 | 0.90 | 0.002 | 0.308 |
|  | Fourth quartile | 0.80 | 0.67 | 0.94 | 0.008 | 0.316 |
| MIP-1α | Second quartile | 0.97 | 0.85 | 1.11 | 0.692 | 0.980 |
|  | Third quartile | 0.87 | 0.76 | 1.00 | 0.052 | 0.443 |
|  | Fourth quartile | 0.99 | 0.87 | 1.14 | 0.939 | 0.980 |
| MIP-1β | Second quartile | 0.97 | 0.77 | 1.21 | 0.763 | 0.980 |
|  | Third quartile | 0.89 | 0.71 | 1.11 | 0.308 | 0.911 |
|  | Fourth quartile | 1.05 | 0.84 | 1.31 | 0.650 | 0.980 |
| PlGF | Second quartile | 1.00 | 0.91 | 1.09 | 0.925 | 0.980 |
|  | Third quartile | 1.03 | 0.94 | 1.12 | 0.586 | 0.980 |
|  | Fourth quartile | 1.04 | 0.95 | 1.14 | 0.377 | 0.918 |
| SAA | Second quartile | 1.28 | 1.04 | 1.58 | 0.020 | 0.335 |
|  | Third quartile | 1.31 | 1.06 | 1.62 | 0.012 | 0.316 |
|  | Fourth quartile | 1.20 | 0.98 | 1.48 | 0.084 | 0.549 |
| TARC | Second quartile | 0.96 | 0.75 | 1.24 | 0.753 | 0.980 |
|  | Third quartile | 0.92 | 0.71 | 1.18 | 0.495 | 0.967 |
|  | Fourth quartile | 0.82 | 0.64 | 1.05 | 0.112 | 0.622 |
| TLSP | Second quartile | 0.92 | 0.78 | 1.10 | 0.360 | 0.911 |
|  | Third quartile | 0.85 | 0.72 | 1.01 | 0.061 | 0.479 |
|  | Fourth quartile | 0.99 | 0.83 | 1.17 | 0.887 | 0.980 |
| TNF-α | Second quartile | 0.95 | 0.82 | 1.10 | 0.506 | 0.967 |
|  | Third quartile | 0.91 | 0.79 | 1.05 | 0.211 | 0.811 |
|  | Fourth quartile | 0.92 | 0.80 | 1.06 | 0.258 | 0.875 |
| TNF-β | Second quartile | 0.97 | 0.92 | 1.02 | 0.269 | 0.897 |
|  | Third quartile | 1.00 | 0.95 | 1.05 | 0.980 | 0.995 |
|  | Fourth quartile | 0.99 | 0.94 | 1.04 | 0.641 | 0.980 |
| Tie-2 | Second quartile | 1.04 | 0.96 | 1.12 | 0.347 | 0.911 |
|  | Third quartile | 0.98 | 0.91 | 1.06 | 0.622 | 0.980 |
|  | Fourth quartile | 1.03 | 0.95 | 1.12 | 0.429 | 0.957 |
| VEGF-A | Second quartile | 0.98 | 0.76 | 1.25 | 0.866 | 0.980 |
|  | Third quartile | 1.08 | 0.84 | 1.38 | 0.564 | 0.980 |
|  | Fourth quartile | 0.92 | 0.72 | 1.18 | 0.507 | 0.967 |
| VEGF-C | Second quartile | 0.95 | 0.84 | 1.06 | 0.351 | 0.911 |
|  | Third quartile | 0.99 | 0.89 | 1.12 | 0.918 | 0.980 |
|  | Fourth quartile | 0.98 | 0.87 | 1.09 | 0.690 | 0.980 |
| VEGF-D | Second quartile | 0.97 | 0.85 | 1.10 | 0.631 | 0.980 |
|  | Third quartile | 1.03 | 0.90 | 1.18 | 0.640 | 0.980 |
|  | Fourth quartile | 0.99 | 0.87 | 1.13 | 0.936 | 0.980 |
| bFGF | Second quartile | 1.03 | 0.79 | 1.33 | 0.827 | 0.980 |
|  | Third quartile | 0.95 | 0.74 | 1.24 | 0.723 | 0.980 |
|  | Fourth quartile | 0.76 | 0.59 | 0.99 | 0.040 | 0.404 |
| sICAM-1 | Second quartile | 1.06 | 0.96 | 1.17 | 0.232 | 0.850 |
|  | Third quartile | 0.95 | 0.86 | 1.05 | 0.360 | 0.911 |
|  | Fourth quartile | 1.08 | 0.97 | 1.19 | 0.149 | 0.720 |
| sVCAM-1 | Second quartile | 1.04 | 0.95 | 1.15 | 0.403 | 0.936 |
|  | Third quartile | 0.97 | 0.88 | 1.07 | 0.597 | 0.980 |
|  | Fourth quartile | 1.08 | 0.98 | 1.19 | 0.102 | 0.603 |
| Abbreviations: CI, confidence interval, FDR, false discovery rate | | | | | | |

## Supplementary Figures

Supplementary Figure 1


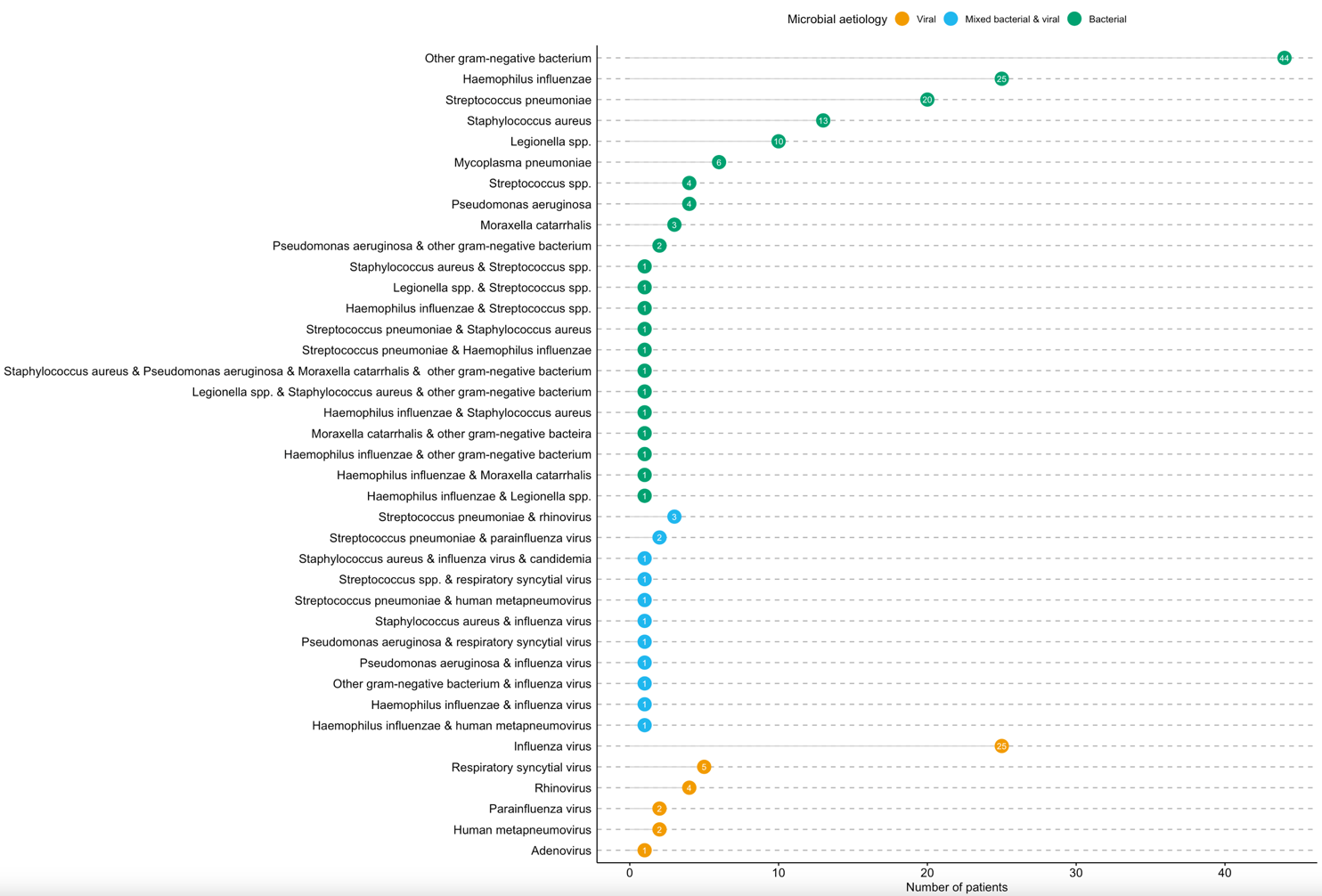


**Supplementary Figure 1:** Figure of isolated pathogens with the x-axis denoting the number of patients from which the pathogens were isolated. Other gram-negative bacteria encompass isolation of *Escherichia coli, Klebsiella pneumoniae, K. oxytoca, Enterobacter* spp.*, Stenotrophomonas maltophilia, Eikenella corrodens, Neisseria meningitidis, Proteus* spp. or *P*. *mirabilis*.

Supplementary Figure 2


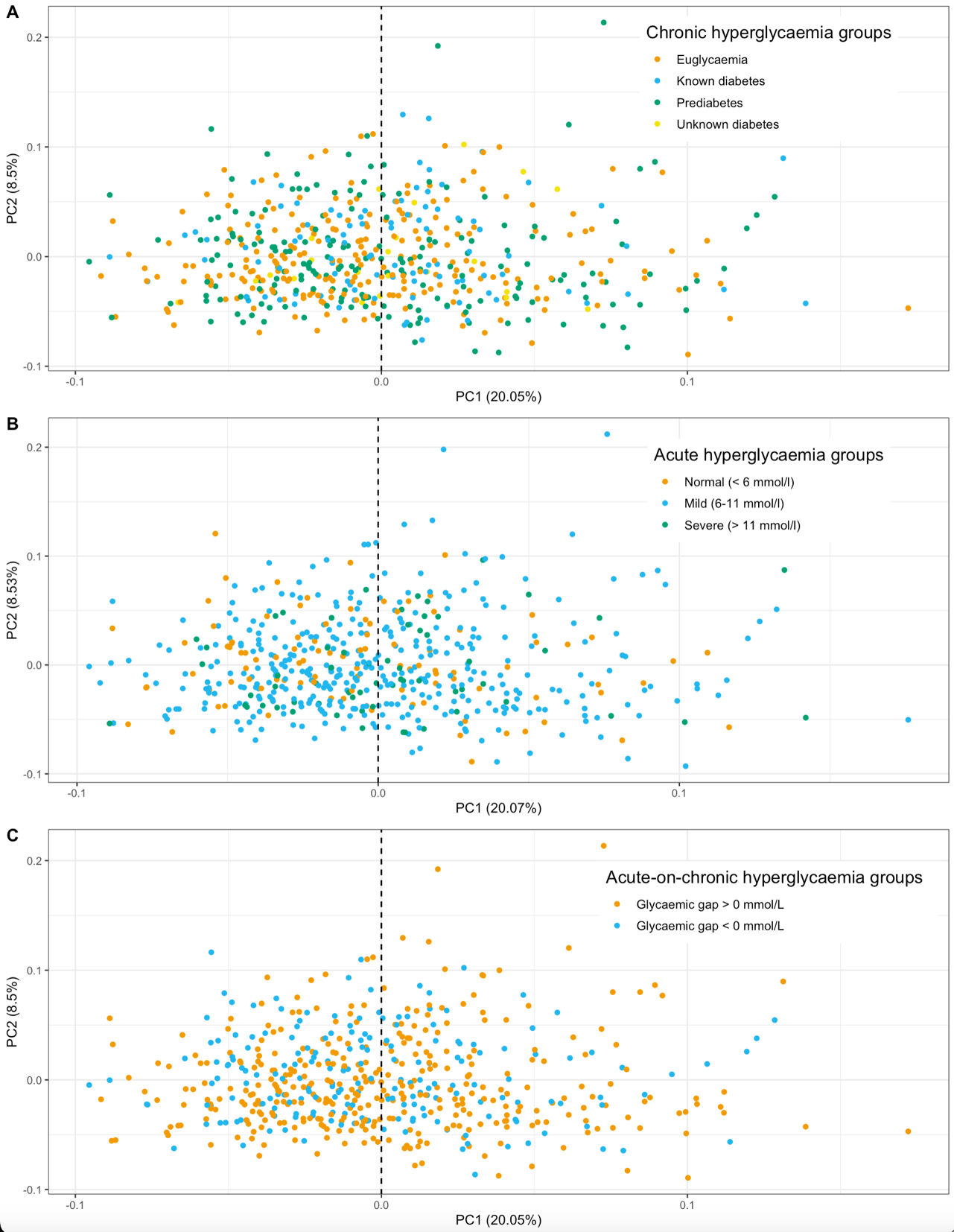


**Supplementary Figure 2:** This figure shows plots of the principal component analysis for all biomarkers. The X-axis shows the percentage of explained variance on principal component (PC) 1, while the Y-axis shows PC2, stratified by chronic hyperglycaemia groups (A), acute hyperglycaemia groups (C), and acute-on-chronic hyperglycaemia groups (D)

Supplementary Figure 3A


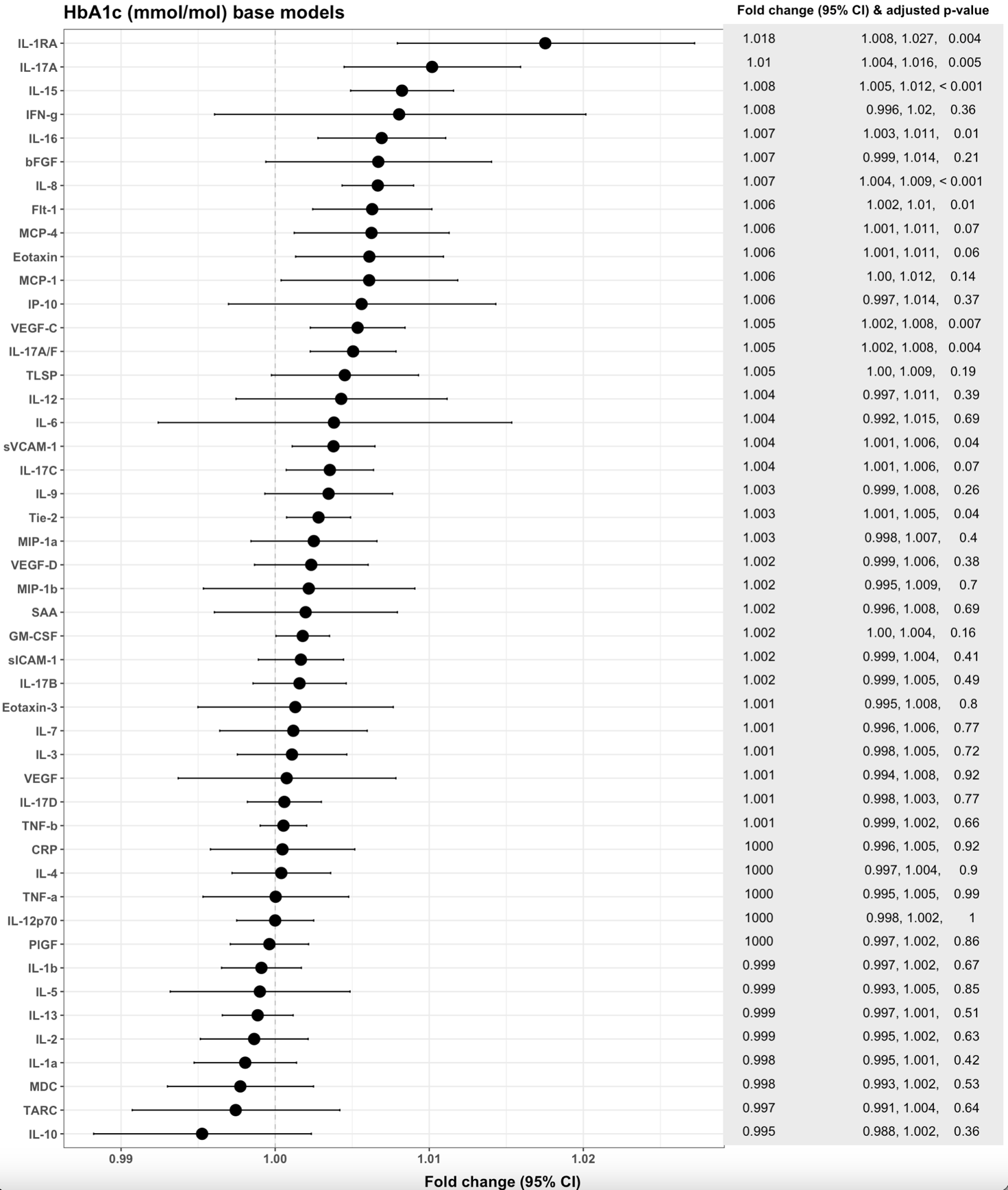


Supplementary Figure 3B


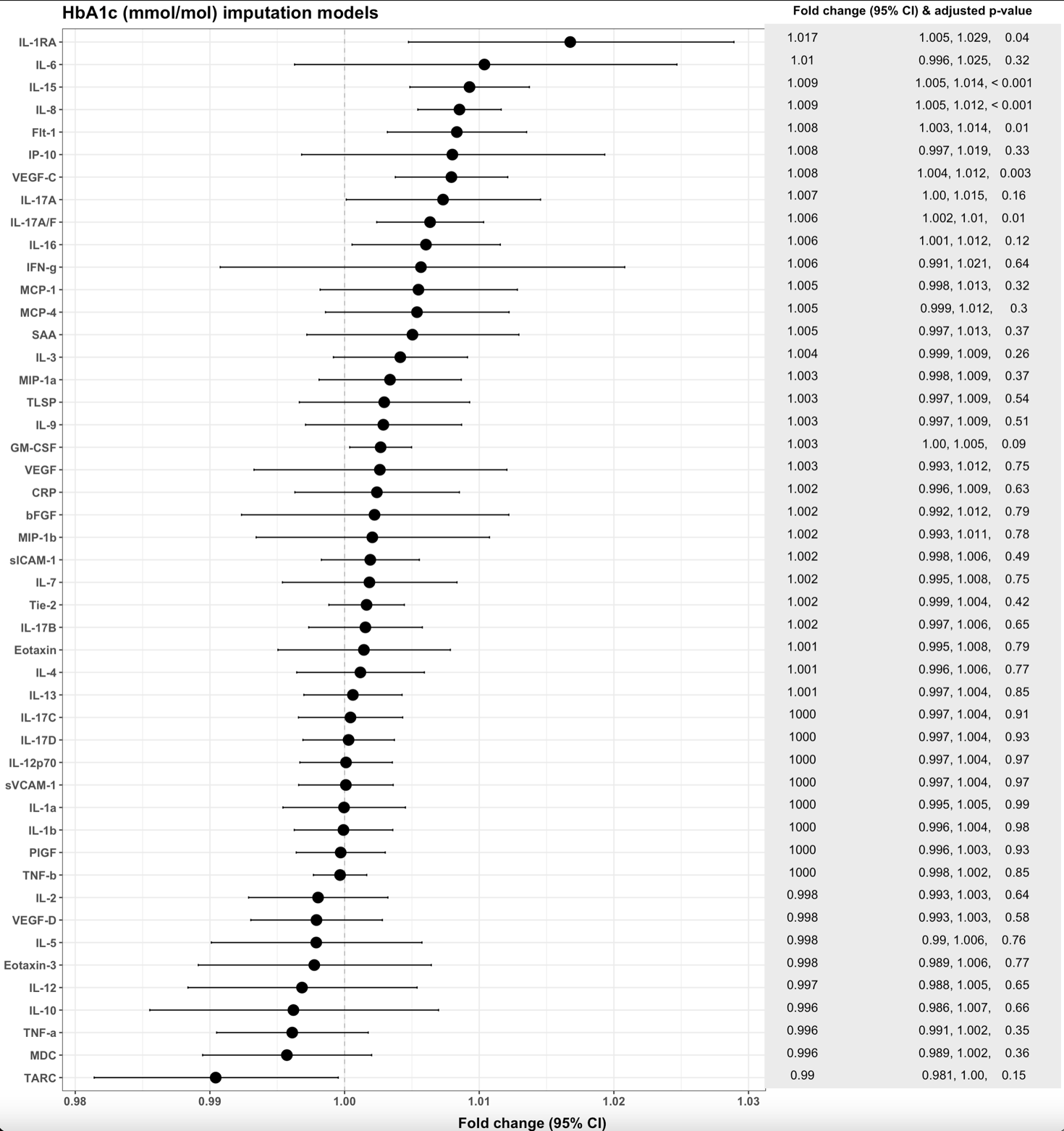


**Supplementary Figure 3:** The figure consists of a forest plot with a table, presenting the main results from the base models (A) and fully adjusted models with imputed data (B) for all biomarkers with HbA1c as the predictor. Estimates are from linear regression models and are reported as the fold change in biomarker concentration for a 1 mmol/mol increase in HbA1c.

Supplementary Figure 4


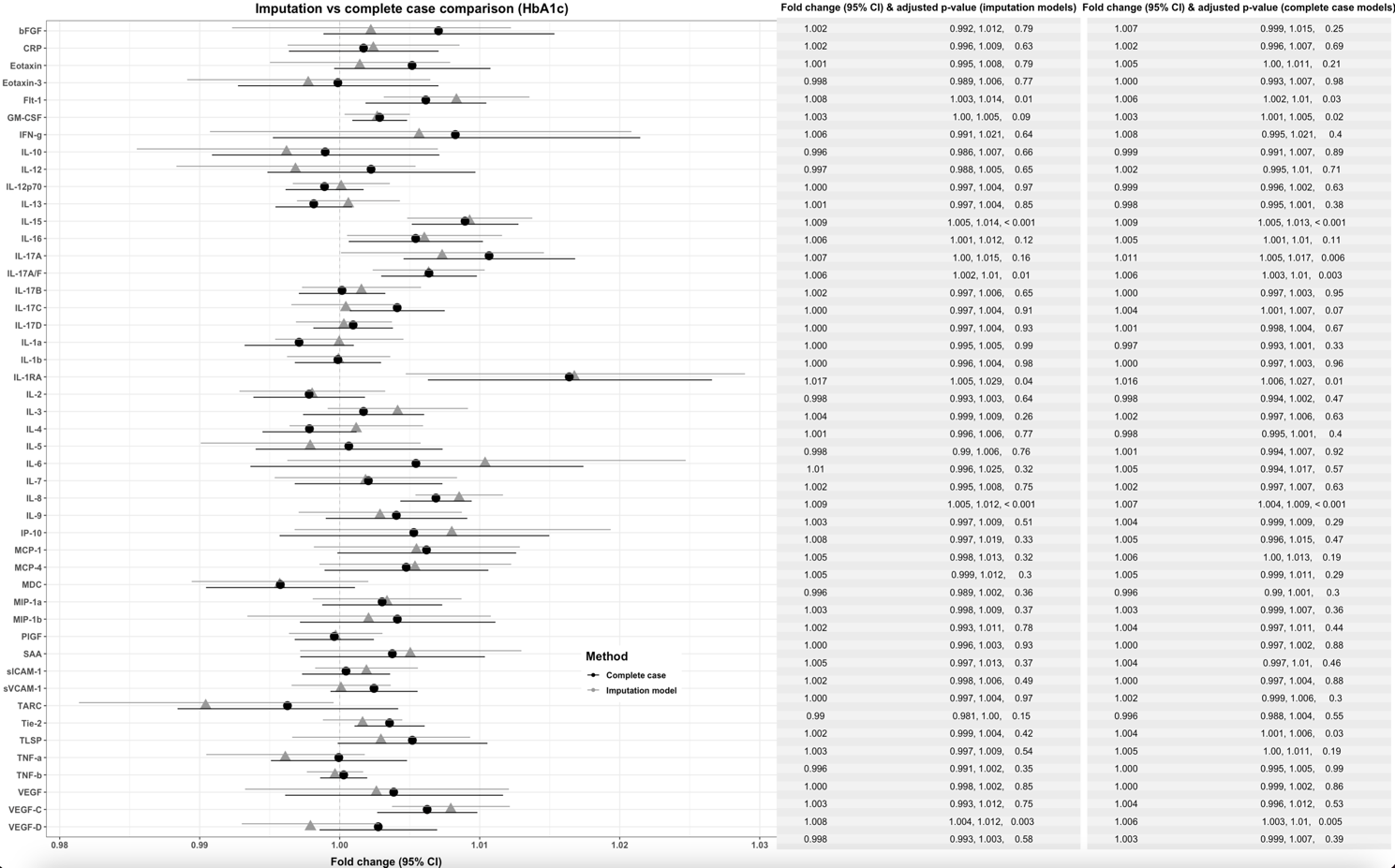


**Supplementary Figure 4:** The figure consists of forest plots with tables, presenting the results from analyses with imputed data and complete case analysis for all biomarkers with HbA1c as the predictor. Estimates are from linear regression models and are reported as the fold change in biomarker concentration for a 1 mmol/mol increase in the HbA1c.

Supplementary Figure 5A


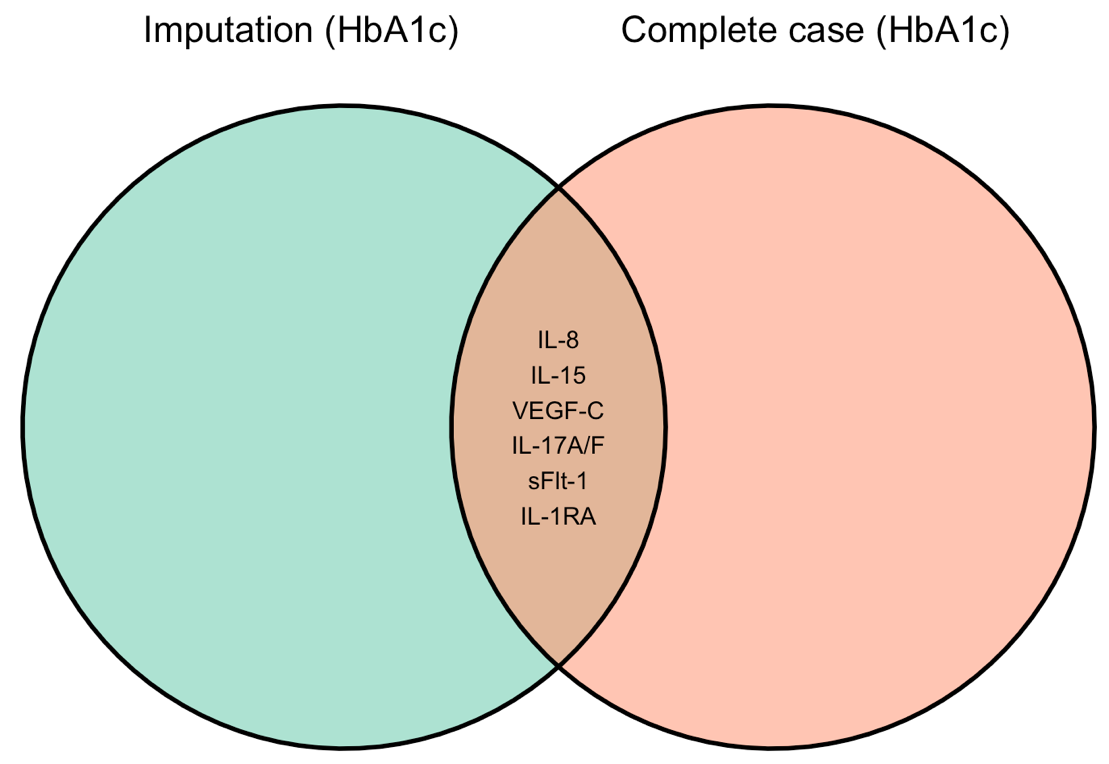


Supplementary Figure 5B


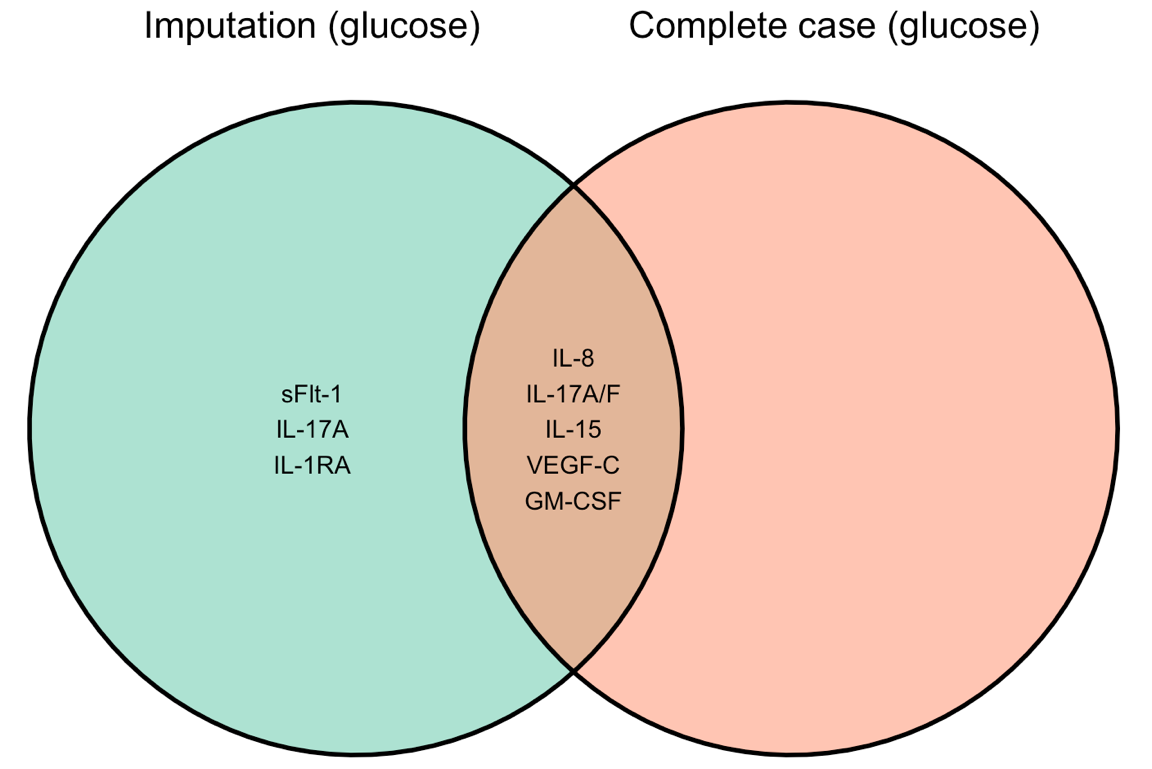


Supplementary Figure 5C


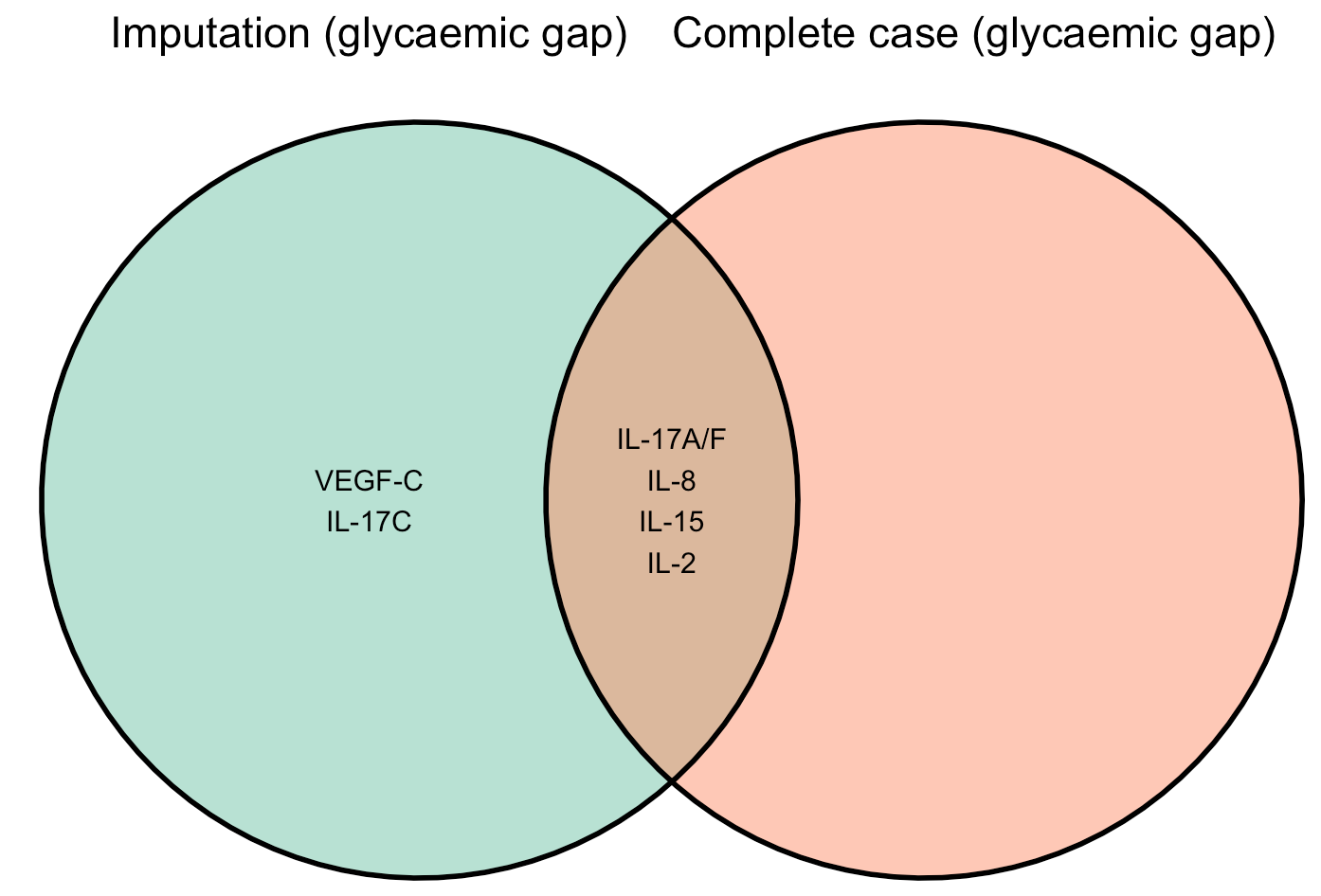


**Supplementary Figure 5**. Venn diagrams of overlapping biomarkers from models with imputed data and complete case analysis with HbA1c (A), admission p-glucose (B) and the glycaemic gap (C) as predictors.

Supplementary Figure 6A


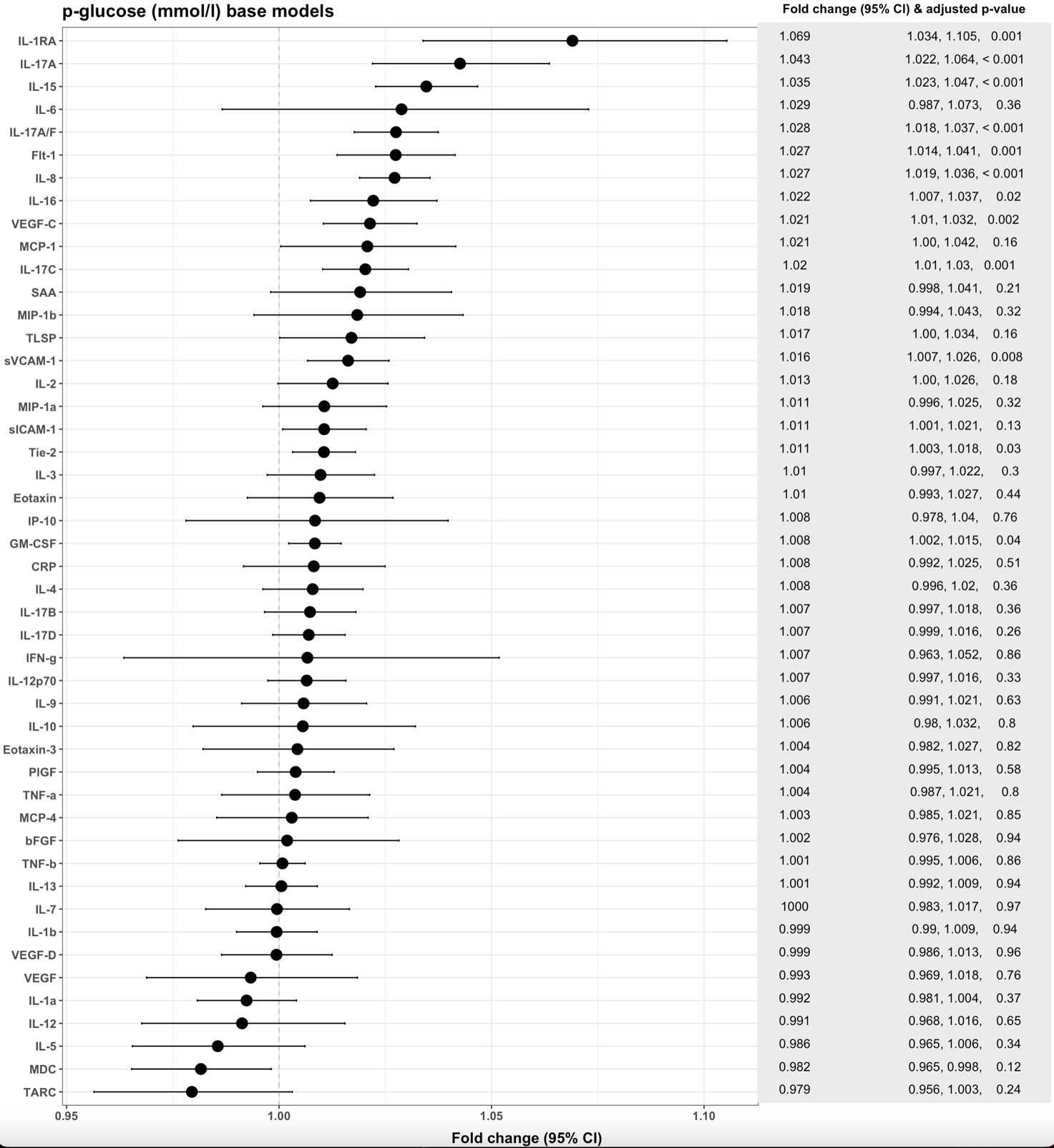


Supplementary Figure 6B


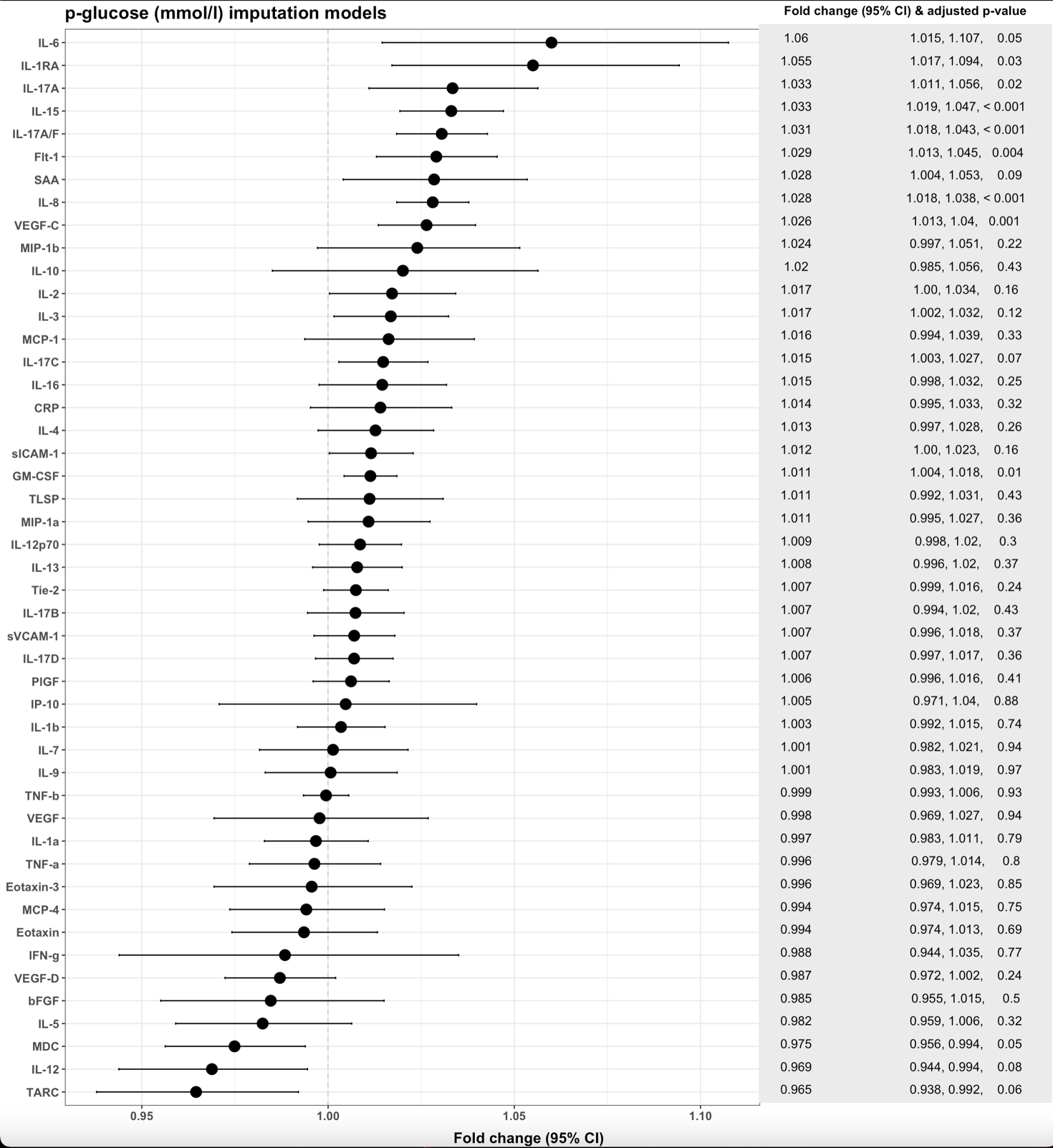


**Supplementary Figure 6:** The figure consists of forest plots with a table, presenting the results from the base models (A) and fully adjusted models with imputed data (B) for all biomarkers with admission p-glucose as the predictor. Estimates are from linear regression models and are reported as the fold change in biomarker concentration for 1 mmol/l increase in p-glucose.

Supplementary Figure 7


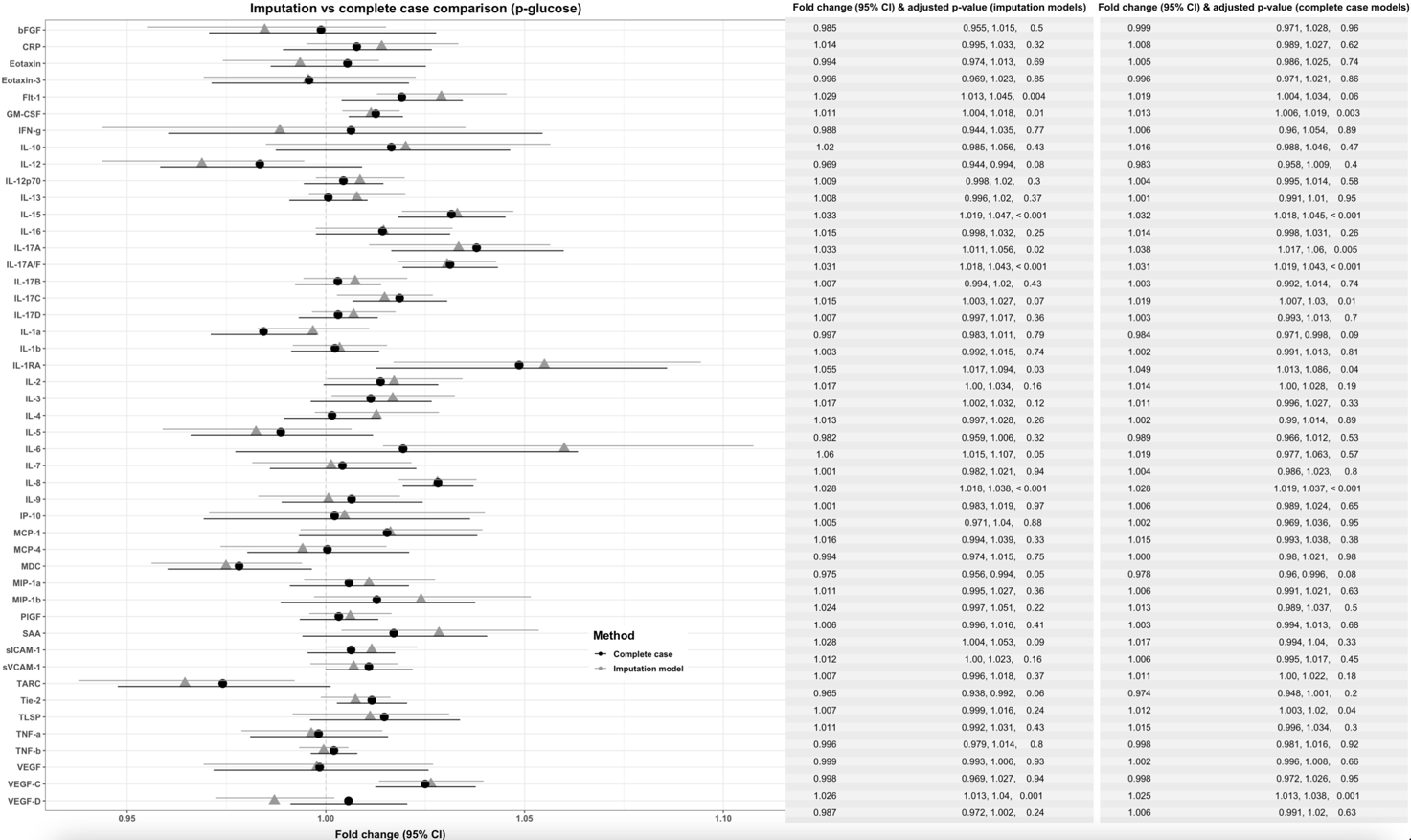


**Supplementary Figure 7:** The figure consists of forest plots with tables, presenting the results from analyses with imputed data and complete case analysis for all biomarkers with admission p-glucose as the predictor. Estimates are from linear regression models and are reported as the fold change in biomarker concentration for 1 mmol/l increase in admission p-glucose.

Supplementary Figure 8A


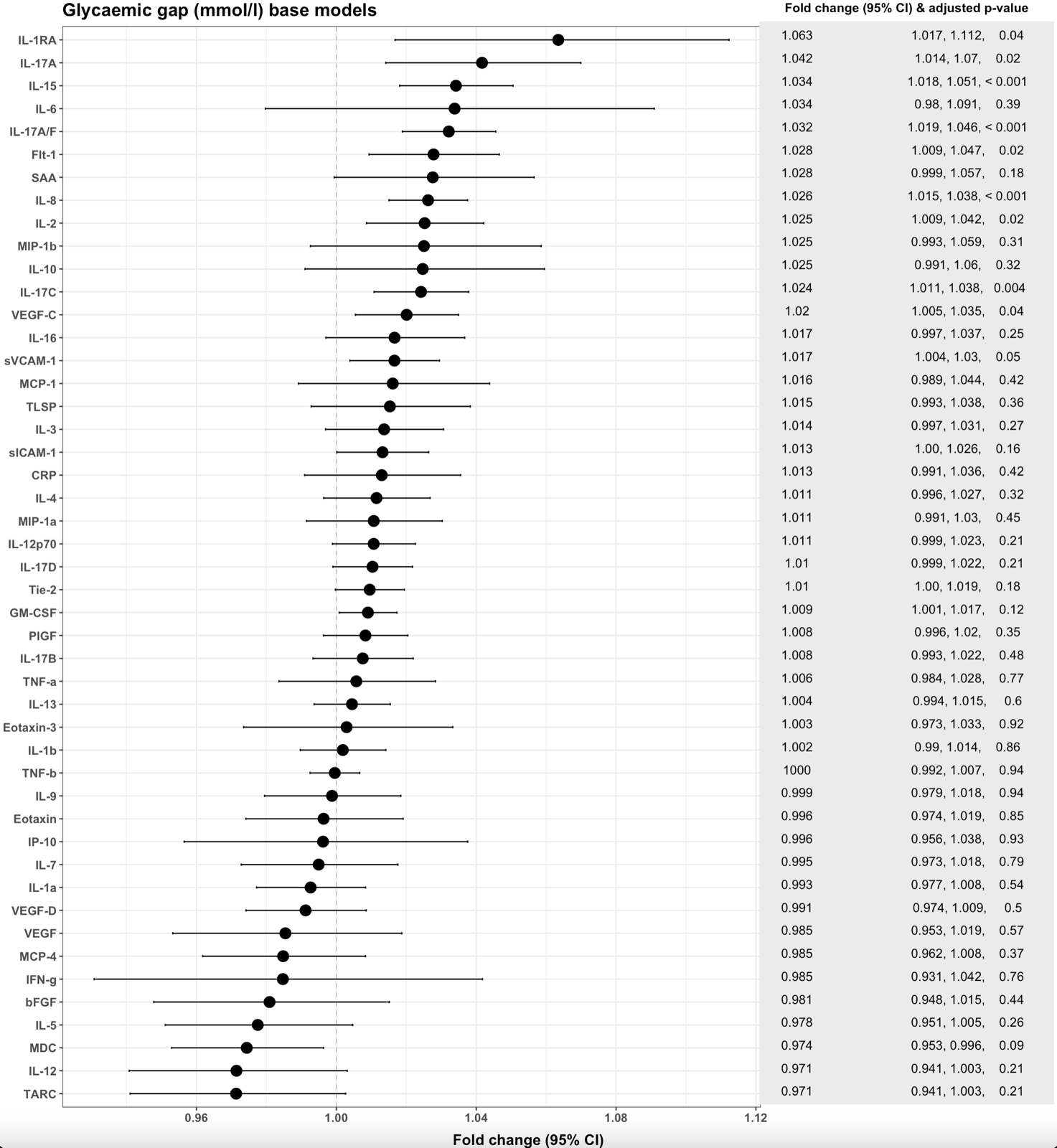


Supplementary Figure 8B


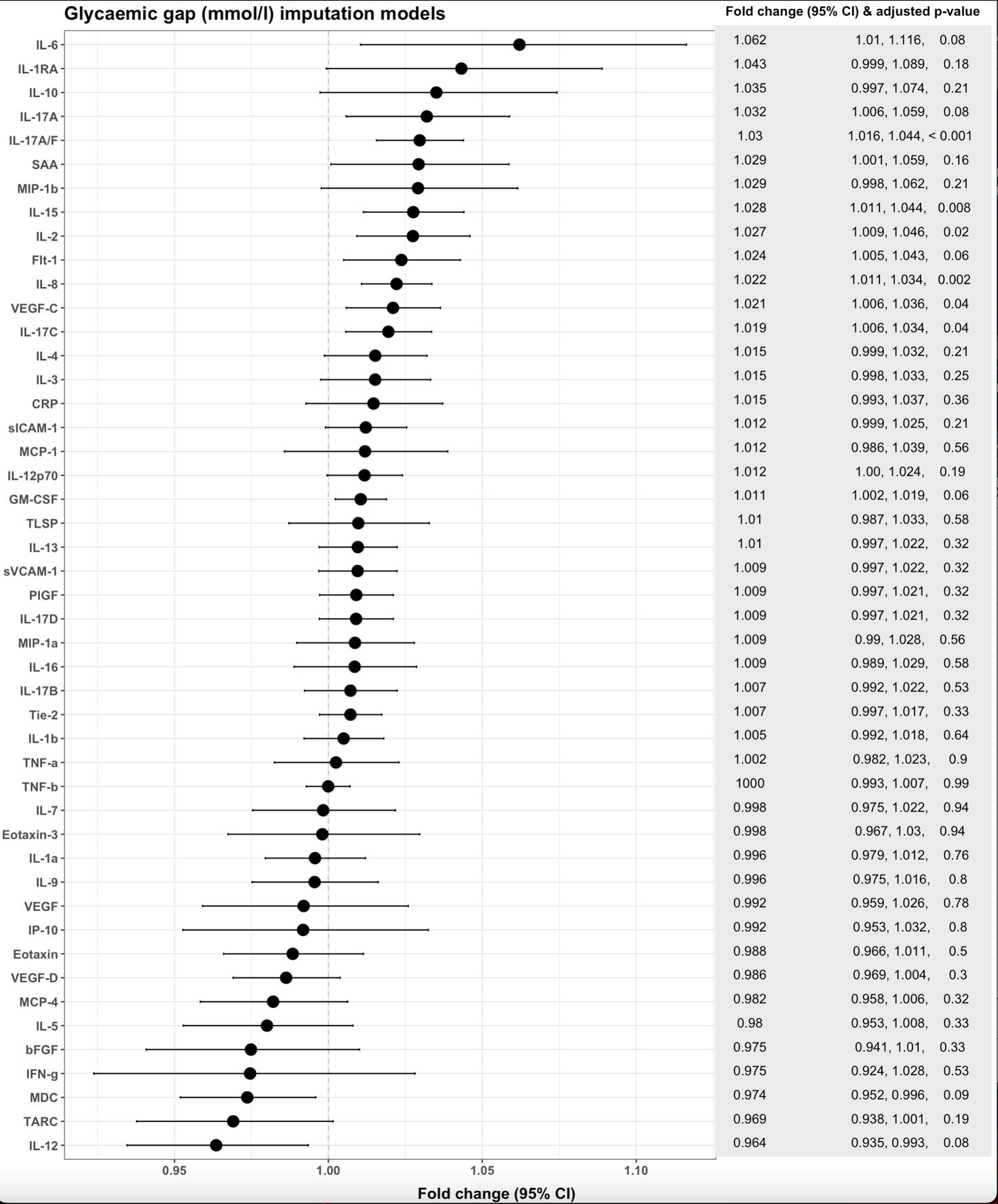


**Supplementary Figure 8:** The figure consists of forest plots with a table, presenting the results from the base models (A) and fully adjusted models with imputed data (B) for all biomarkers with the glycaemic gap as the predictor. Estimates are from linear regression models and are reported as the fold change in biomarker concentration for a 1 mmol/l increase in the glycaemic gap.

Supplementary Figure 9:


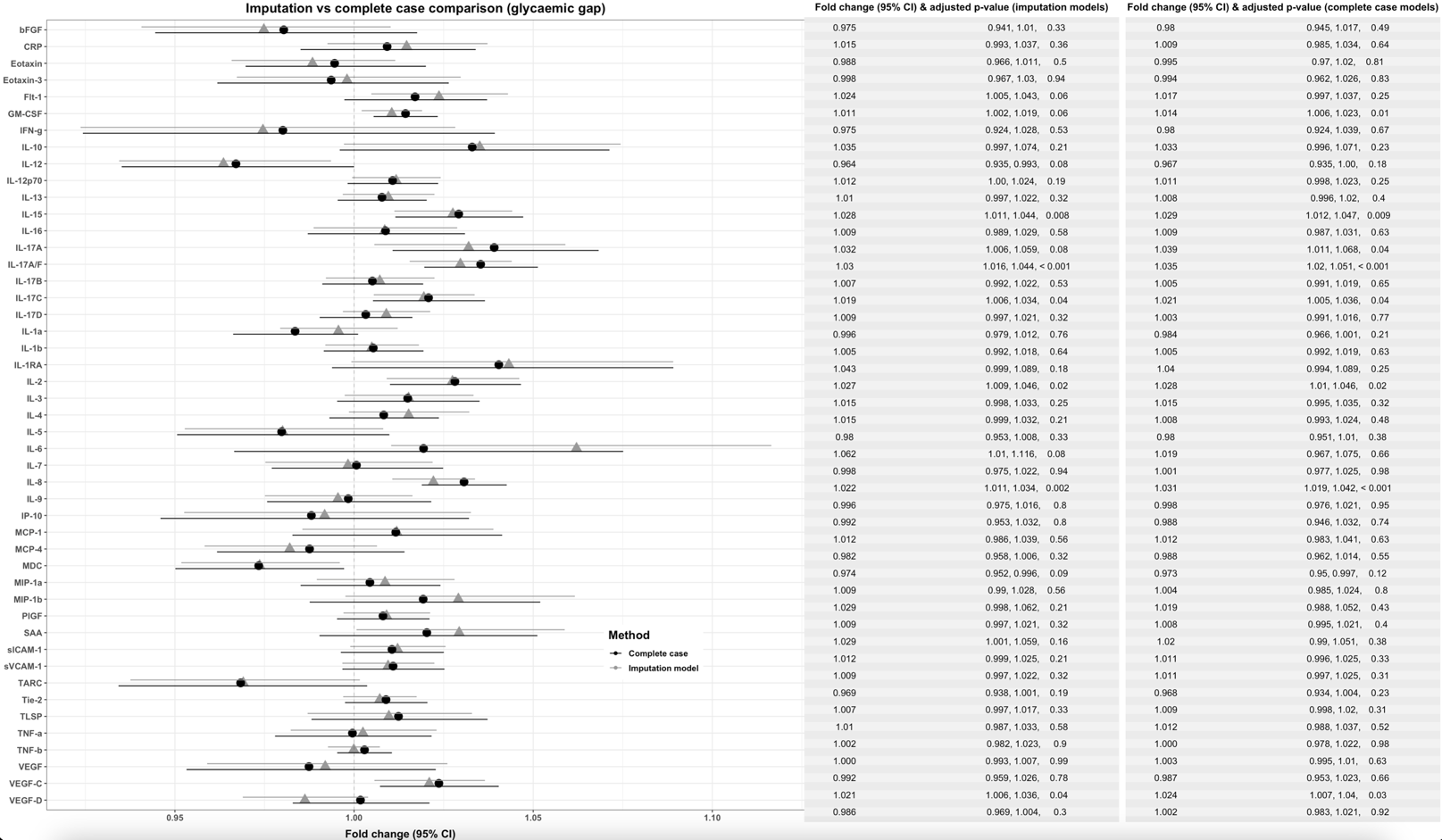


**Supplementary Figure 9:** The figure consists of forest plots with tables, presenting the results from analyses with imputed data and complete case analysis for all biomarkers with the glycaemic gap as the predictor. Estimates are from linear regression models and are reported as the fold change in biomarker concentration for a 1 mmol/l increase in the glycaemic gap.

# References

1. Torres A, Peetermans WE, Viegi G, Blasi F. Risk factors for community-acquired pneumonia in adults in Europe: a literature review. Thorax. 2013 Nov;68(11):1057–65.

2. Sproston NR, Ashworth JJ. Role of C-Reactive Protein at Sites of Inflammation and Infection. Front Immunol. 2018 Apr 13;9:754.

3. Dinarello CA. Historical Review of Cytokines. Eur J Immunol. 2007 Nov;37(Suppl 1):S34–45.

4. Turner MD, Nedjai B, Hurst T, Pennington DJ. Cytokines and chemokines: At the crossroads of cell signalling and inflammatory disease. Biochimica et Biophysica Acta (BBA) - Molecular Cell Research. 2014 Nov 1;1843(11):2563–82.

5. Apte RS, Chen DS, Ferrara N. VEGF in Signaling and Disease: Beyond Discovery and Development. Cell. 2019 Mar 7;176(6):1248–64.

6. Dong C. Cytokine Regulation and Function in T Cells. Annu Rev Immunol. 2021 Apr 26;39:51–76.

7. Pierrakos C, Velissaris D, Bisdorff M, Marshall JC, Vincent JL. Biomarkers of sepsis: time for a reappraisal. Crit Care. 2020 Jun 5;24(1):287.
